# Fluorophore‐Labeled Cyclic Nucleotides as Potent Agonists of Cyclic Nucleotide‐Regulated Ion Channels

**DOI:** 10.1002/cbic.202000116

**Published:** 2020-05-04

**Authors:** Marco Lelle, Maik Otte, Michele Bonus, Holger Gohlke, Klaus Benndorf

**Affiliations:** ^1^ Institute of Physiology II University Hospital Jena Kollegiengasse 9 07743 Jena Germany; ^2^ Institute for Pharmaceutical and Medicinal Chemistry Heinrich Heine University Düsseldorf Universitätsstrasse 1 40225 Düsseldorf Germany; ^3^ John von Neumann Institute for Computing (NIC) Jülich Supercomputing Centre (JSC) and Institute of Biological Information Processing (IBI-7: Structural Biochemistry) Forschungszentrum Jülich GmbH Wilhelm-Johnen-Strasse 52425 Jülich Germany

**Keywords:** fluorescent probes, ion channels, nucleotides

## Abstract

High‐affinity fluorescent derivatives of cyclic adenosine and guanosine monophosphate are powerful tools for investigating their natural targets. Cyclic nucleotide‐regulated ion channels belong to these targets and are vital for many signal transduction processes, such as vision and olfaction. The relation of ligand binding to activation gating is still challenging, and there is a need for fluorescent probes that enable the process to be broken down to the single‐molecule level. This inspired us to prepare fluorophore‐labeled cyclic nucleotides, which are composed of a bright dye and a nucleotide derivative with a thiophenol motif at position 8 that has already been shown to enable superior binding affinity. These bioconjugates were prepared by a novel cross‐linking strategy that involves substitution of the nucleobase with a modified thiophenolate in good yield. Both fluorescent nucleotides are potent activators of different cyclic nucleotide‐regulated ion channels with respect to the natural ligand and previously reported substances. Molecular docking of the probes excluding the fluorophore reveals that the high potency can be attributed to additional hydrophobic and cation‐π interactions between the ligand and the protein. Moreover, the introduced substances have the potential to investigate related target proteins, such as cAMP‐ and cGMP‐dependent protein kinases, exchange proteins directly activated by cAMP or phosphodiesterases.

## Graphical abstract

**Selecting channels**: Fluorescent cyclic nucleotide derivatives synthesized with a thiophenol motif‐containing heterobifunctional cross‐linking reagent efficiently activate CNG as well as HCN channels in the nanomolar range and are potential candidates to study related target proteins.

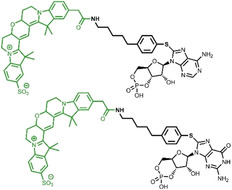

## Introduction

Fluorescent probes are indispensable compounds to study the structure and function of proteins as well as associated biological processes.^1^ Among these compounds, fluorophore‐labeled derivatives of cyclic adenosine and guanosine monophosphate (cAMP and cGMP) play an undisputed role to investigate cAMP‐ and cGMP‐dependent protein kinases, exchange proteins directly activated by cAMP and, in particular, cyclic nucleotide‐regulated ion channels.^2^ These targets comprise cyclic nucleotide‐gated (CNG) and hyperpolarization‐activated cyclic nucleotide‐modulated (HCN) channels, both belonging to the superfamily of tetrameric cyclic nucleotide‐regulated ion channels.^3^ Despite homologue sequences, the function of these two classes of channels is remarkably different. CNG channels play an essential role in the signal transduction of the olfactory and visual system, whereas HCN channels generate electrical rhythmicity in specialized neurons and cardiomyocytes.^4^ Both types of channels respond to the binding of cyclic nucleotides to a tetrameric cyclic nucleotide‐binding domain (CNBD). However, in contrast to CNG channels, HCN channels require a sufficiently hyperpolarizing membrane voltage as primary activating stimulus.

**Figure 1:**
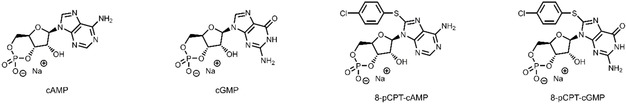
Chemical structures of cyclic nucleotide‐regulated ion channel agonists.

The CNBD can accommodate and tolerate cyclic nucleotides with large substituents in 8‐position, while other modifications of the cyclic nucleotide often impair channel activation.^5^ An efficient fluorescent ligand to investigate cyclic nucleotide‐regulated ion channels should have a high affinity towards the receptor and should be a full agonist, that is, have a high potency. The 8‐substituted analogs of cAMP and cGMP 8‐(4‐chlorophenylthio)adenosine‐3’,5’‐cyclic monophosphate (8‐pCPT‐cAMP) as well as 8‐(4‐chlorophenylthio)guanosine‐3’,5’‐cyclic monophosphate (8‐pCPT‐cGMP) are known to have these desired properties, which renders their modification with a fluorescent dye attractive (Figure 1).^6^ However, the thiophenol‐substituted cyclic nucleotides have never been further modified. Dye conjugates composed of a bright fluorophore and the 8‐substituted derivatives should be powerful tools to relate ligand binding to activation gating in cyclic nucleotide‐regulated ion channels.

In this work, we introduce the synthesis of fluorophore‐labeled derivatives of 8‐pCPT‐cAMP and 8‐pCPT‐cGMP. The dye conjugates were prepared with a novel heterobifunctional cross‐linking reagent containing the thiophenol motif. To evaluate the potency of the synthesized fluorescent probes, the effect of the compounds on different cyclic nucleotide‐regulated ion channels was examined and compared to cAMP as well as cGMP by studying the activation of ion channels with the patch‐clamp technique. Ligand binding and channel activation for the most potent fluorescent agonist was measured in parallel by confocal patch‐clamp fluorometry.2a To get deeper insights how the derivatives evolve their affinity, a chemical approach, including the preparation of molecules lacking the sulfur atom, was carried out, and the results were interpreted based on results from molecular docking.

## Results and Discussion

### Preparation of cyclic nucleotide derivatives

**Scheme 1:**
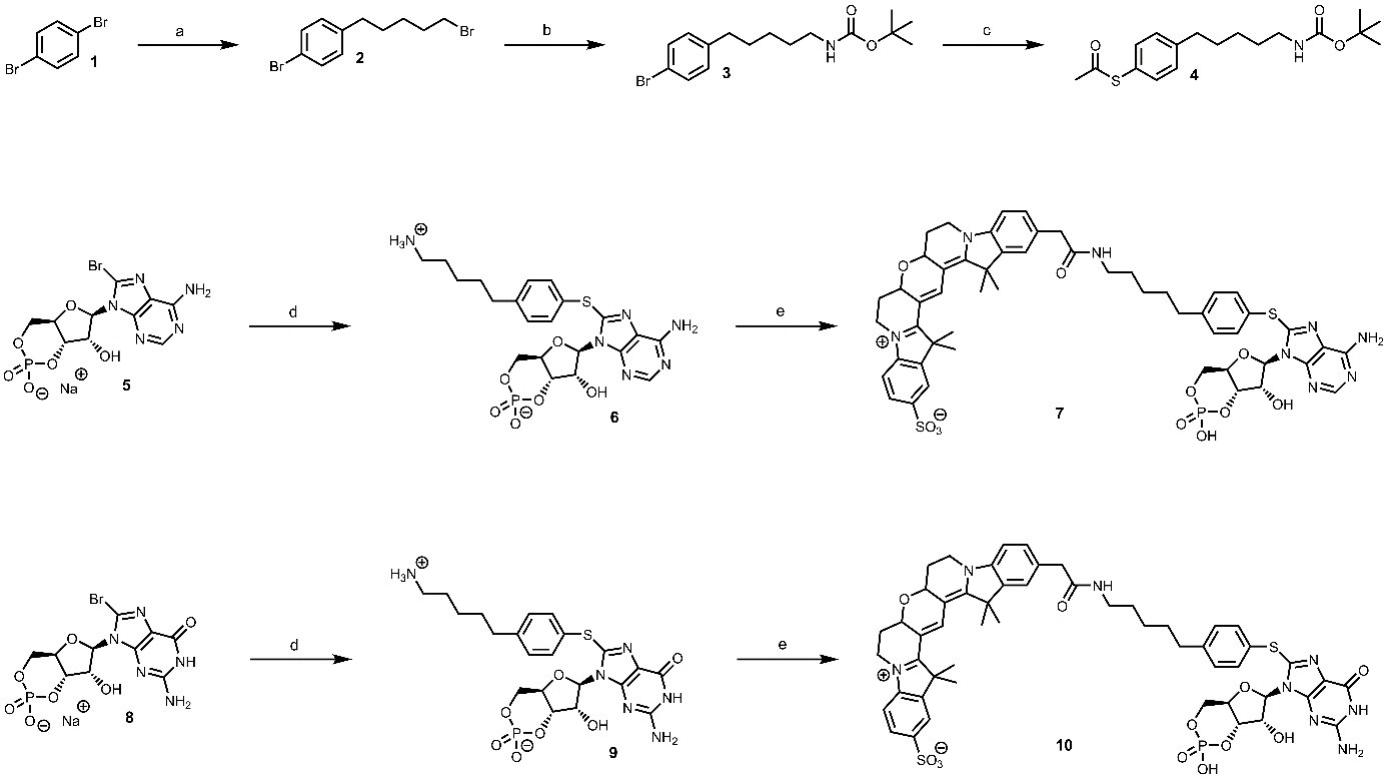
Synthesis of fluorescent cyclic nucleotide derivatives by use of a thiophenol motif‐containing heterobifunctional cross‐linking reagent. (a) 1. *n*‐butyllithium (1 equiv.), dry tetrahydrofuran, argon, −78 °C, 30 min 2. 1,5‐dibromopentane (3 equiv.), argon, −78 °C to room temperature, 2 h, 59 %; (b) 1. 7 M ammonia, methanol, 100 °C, 1 h 2. di‐*tert*‐butyl dicarbonate (1 equiv.), triethylamine (2 equiv.), dry tetrahydrofuran, argon, 50 °C, 2 h, 83 %; (c) potassium thioacetate (1.2 equiv.), Pd_2_(dba)_3_ (0.05 equiv.), Xantphos (0.1 equiv.), dry 1,4‐dioxane, argon, 80 °C, 24 h, 72 %; (d) 1. **4** (1.5 equiv.), 0.1 M sodium hydroxide solution/1,4‐dioxane 1 : 1, argon, 70 °C, 4 h 2. 2 M hydrochloric acid, 70 °C, 2 h, 68 % **6**, 53 % **9**; (e) nucleotide derivative (1.1 equiv.), Cy3B NHS ester (1 equiv.), triethylamine (20 equiv.), dry *N*,*N*‐dimethylformamide, argon, room temperature, overnight, 44 % **7**, 52 % **10**.

Fluorophore‐labeled cyclic nucleotides were synthesized with a novel heterobifunctional cross‐linking reagent, which can react with 8‐bromo‐substituted purine nucleobases as well as active esters of fluorescent dyes (Scheme 1). To prepare the thiophenol motif‐containing crosslinker, 1,4‐dibromobenzene was initially modified with an alkyl chain. The hydrocarbon functions as a spacer between fluorophore and cyclic nucleotide. Thereafter, an amino group was introduced onto the alkyl chain with ammonia and subsequently protected with the acid‐labile Boc group, to avoid side reactions in further synthesis steps. The other bromo substituent of the benzene ring was transferred with potassium thioacetate into a thioester, which typically requires homogenous catalysis with tris(dibenzylideneacetone)dipalladium(0) (Pd_2_(dba)_3_) and 4,5‐bis(diphenylphosphino)‐9,9‐dimethylxanthene (Xantphos) as ligand due to the low reactivity towards nucleophiles. The obtained aryl thioacetate **4** is the desired cross‐linking reagent and carries a protected sulfhydryl group. This thiol is easily accessible in the presence of base and was reacted with the 8‐bromo derivatives of cAMP and cGMP (**5**, **8**). Subsequent deprotection of the amino group yielded cyclic nucleotides that carry the thiophenol motif, which has an amino‐functionalized alkyl chain in 4‐position. These nucleotide derivatives were modified with Cy3B by the *N*‐hydroxysuccinimide ester of the dye, to yield the fluorescent probes **7** and **10** after chromatographic purification. The restrained cyanine dye was chosen over other fluorophores because of its outstanding properties, such as exceptional brightness, which is beneficial for labeling and imaging applications.^7^

**Scheme 2:**
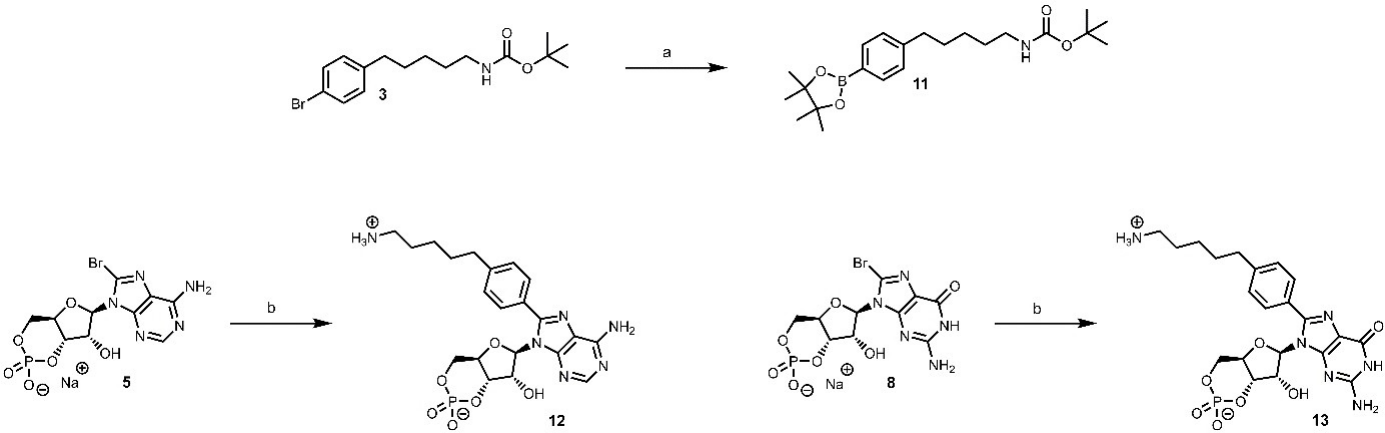
Synthesis of rigidified cAMP and cGMP derivatives without sulfur atom. (a) bis(pinacolato)diboron (1.3 equiv.), Pd(dppf)Cl_2_ (0.15 equiv.), potassium acetate (3 equiv.), 1,4‐dioxane, argon, 80 °C, 4 h, 86 %; (b) 1. **11** (1.5 equiv.), sSPhos Pd G2 (0.1 equiv.), potassium phosphate (3 equiv.), water/1,4‐dioxane (1 : 1), argon, 70 °C, 4 h 2. 2 M hydrochloric acid, 70 °C, 2 h, 64 % **12**, 7 % **13**.

In our previous study, we revealed that hydrophobicity is a crucial prerequisite to achieve a high affinity of the ligand towards the receptor.5c To investigate how size and, in particular, flexibility of the ligand can influence the activation of ion channels, the corresponding more rigid cyclic nucleotide derivatives without sulfur atom were synthesized (Scheme 2). Therefore, another linking reagent (**11**) was prepared, which carries a boronic ester to enable palladium‐catalyzed cross‐coupling reactions between **11** and the brominated cyclic nucleotides. The crosslinker was synthesized by Miyaura borylation from **3** with [1,1′‐bis(diphenylphosphino)ferrocene]palladium(II) dichloride (Pd(dppf)Cl_2_) as catalyst. Afterwards, the Suzuki reaction was performed by using the water‐soluble Buchwald palladacycle precatalyst chloro(sodium‐2‐dicyclohexylphosphino‐2′,6′‐dimethoxy‐1,1′‐biphenyl‐3′‐sulfonate)[2‐(2′‐amino‐1,1′‐biphenyl)]palladium(II) (sSPhos Pd G2). These catalyst systems containing dialkylbiaryl phosphine ligands have already shown superior properties in Suzuki cross‐couplings.^8^ Nevertheless, the preparation of the cGMP analog **13** was very challenging because of its poor solubility, which is accompanied by this zwitterionic substances and decreased the yield below 10 %.

### Activation of CNG and HCN channels

**Figure 2:**
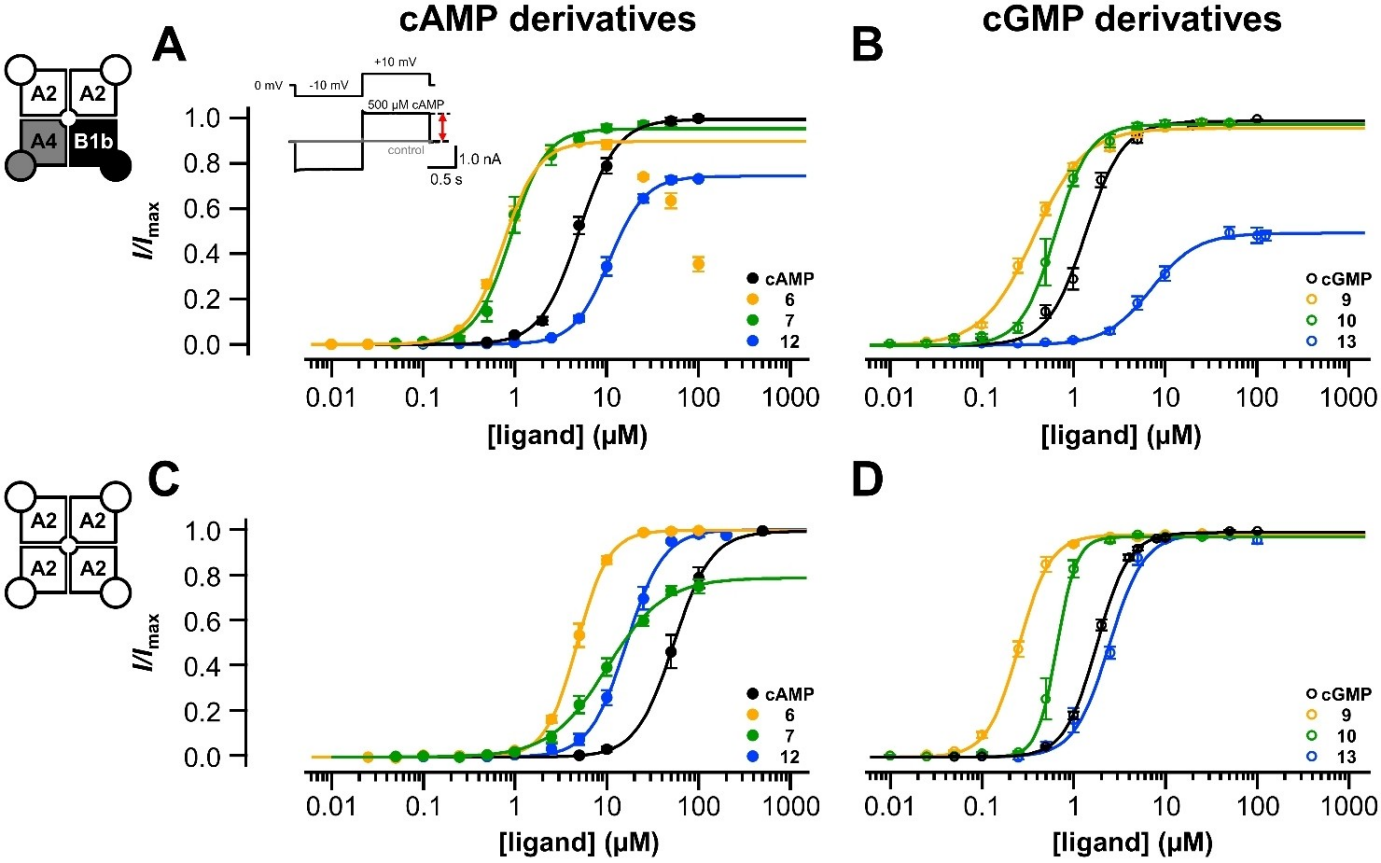
Effect of different ligands on heterotetrameric CNGA2:A4:B1b (A, B) and homotetrameric CNGA2 (C, D) channels, displayed as concentration‐activation relationships. The obtained data points were fitted with Equation 1 (Experimental Section), to give the *EC*_50_ and *H*_a_ values provided in Table 1. The currents were measured following the voltage protocol in A.

We investigated the effects of the novel cyclic nucleotide derivatives on different types of cyclic nucleotide‐regulated ion channels by employing the patch‐clamp technique and compared the potency to that of cAMP and cGMP. First, the effect of the compounds on olfactory CNG channels with natural composition was tested. These channels are composed of two CNGA2 subunits, one CNGA4 subunit and one CNGB1b subunit.^9^ The currents were measured in inside‐out patches at +10 mV, according to the voltage protocol displayed in the inset of Figure 2A. Like the natural ligand cAMP, all substances produced robust currents. To determine the potency of the ligands, full concentration‐activation relationships were generated. These relationships were fitted with Equation 1 (Experimental Section), yielding the concentration of half maximum activation (*EC*_50_) and the Hill coefficient (*H*_a_). The current maxima were normalized with respect to those at saturating cAMP (500 μM) or cGMP (100 μM). Both natural cyclic nucleotides efficiently activated the heterotetrameric channels in the low micromolar range, which is in good agreement with the literature.5b, 5c, 6a The thiophenol‐substituted cAMP derivative without fluorophore (**6**) showed an enhanced apparent affinity compared to the natural agonist. Notably, elevated concentrations led to an inhibitory effect. This effect was not visible for the Cy3B‐functionalized compound, but the potency with a nanomolar affinity was preserved (Figure 2A). In contrast to the sulfur atom‐containing derivatives, **12** was less affine than cAMP and, compared to the other substances, it was only a partial agonist. A similar trend was also observed for the corresponding cGMP derivatives (Figure 2B). The apparent affinity of the dye conjugate was 630 nM (Table 1), which is more potent than many of the previously reported compounds that were used to study CNG and HCN channels (e. g., *EC*_50_=2.1 μM, Otte et al.).^10^

**Table 1:** *EC*_50_ values of the different ligands and Hill coefficients derived from the concentration‐activation relationships in CNG channels (n indicates the number of measurements).

Compound	Heterotetrameric CNGA2:A4:B1b channels			Homotetrameric CNGA2 channels		
	*EC*_50_ [μM]	*H*_a_	n	*EC*_50_ [μM]	*H*_a_	n
cAMP	4.98±0.15	2.08±0.12	7	53.1±0.6	2.12±0.06	4
cGMP	1.36±0.07	2.11±0.23	8	1.77±0.02	2.48±0.05	16
**6**	0.75±0.01	2.25±0.08	7	4.72±0.04	2.51±0.05	9
**7**	0.90±0.04	2.26±0.29	5	10.0±0.5	1.39±0.07	6
**9**	0.37±0.01	1.55±0.08	8	0.25±0.01	2.52±0.08	8
**10**	0.63±0.02	2.32±0.16	6	0.65±0.01	4.03±0.12	8
**12**	10.7±0.1	2.23±0.05	6	16.3±0.5	2.14±0.10	7
**13**	7.02±0.26	1.74±0.11	7	2.41±0.15	2.28±0.28	7

Moreover, we studied the effect of the nucleotide derivatives on homotetrameric CNGA2 channels. These channels are known to be significantly less sensitive to the natural ligand cAMP, as compared to heterotetrameric channels.^11^ Similarly, all synthesized cAMP derivatives were more potent than the natural cyclic nucleotide. However, **7** did not generate maximum activation at 100 μM and the Hill coefficient was exceptionally small (1.39, Table 1). In contrast, the cGMP derivatives were fully efficient and the thiophenol motif‐containing moieties (**9**, **10**) were potent agonists with *EC*_50_ values in the nanomolar range (Figure 2D). Both substances were more potent than cGMP and established fluorophore‐labeled cyclic nucleotide derivatives (e. g., *EC*_50_=1.64 μM, Biskup et al.) in homotetrameric channels, which was not observed for the more rigid molecule lacking the sulfur atom.2a, 10

**Figure 3:**
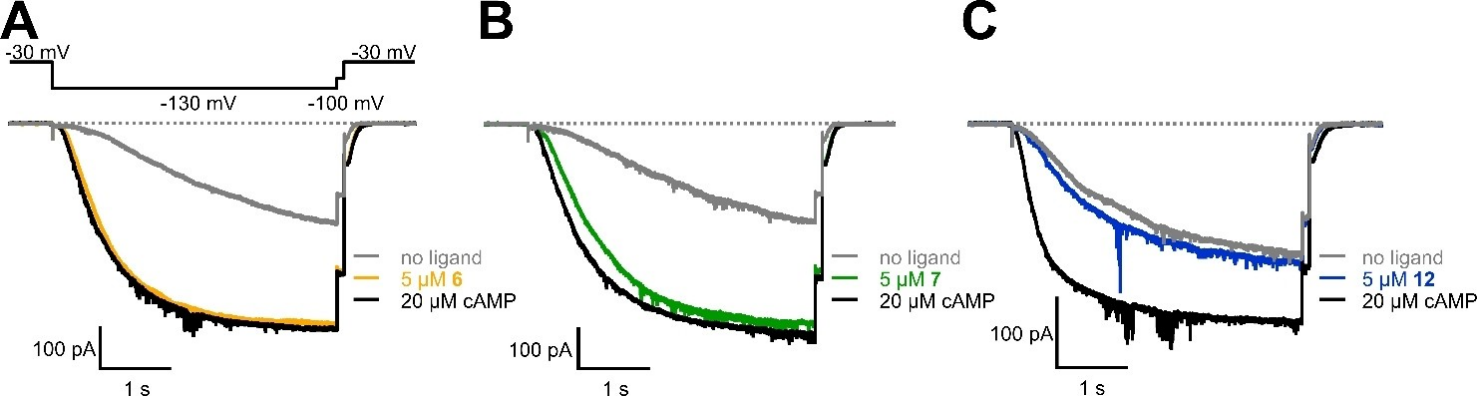
Effect of the cAMP derivatives **6**, **7** and **12** on homotetrameric HCN2 channels. (A, B) Thiophenol‐substituted molecules activated the channels in a similar way to the natural agonist by both accelerating the activation speed and increasing the current amplitude at the end of hyperpolarizing pulses. (C) In contrast, **12** was almost ineffective.

We also tested the ability of the cAMP‐derived substances to activate structurally related homotetrameric HCN2 channels. On these channels the potency of cGMP and its derivatives is negligibly small. These measurements were performed in inside‐out patches as well. The channels were activated from a holding potential of −30 mV by a hyperpolarizing voltage pulse to −130 mV, followed by a short pulse to −100 mV, as described in the inset of Figure 3A. All recordings were carried out in the absence of a ligand at first and then with a solution containing 5 μM of the cyclic nucleotide to be tested. The results were compared to the effect of saturating cAMP (20 μM). Like cAMP, the thiophenol‐substituted cAMP derivative without fluorophore (**6**) accelerated the current and enhanced the amplitude at the end of the hyperpolarizing pulse (Figure 3A). As for CNG channels, the dye conjugate **7** showed a similar effect to that of cAMP (Figure 3B). The efficiency pattern was also preserved for the cAMP derivative lacking the sulfur atom: 5 μM of **12** had no effect (Figure 3C), which confirms again the importance of the sulfur atom for the effect of the compound.

### Relating ligand binding to activation gating in CNG channels

The relation between ligand binding and receptor activation is important for understanding the function of receptors. Therefore, confocal patch‐clamp fluorometry (cPCF) can provide valuable information as shown previously for CNG, HCN and, recently, P2X2 channels.2a, 2b, 12

**Figure 4:**
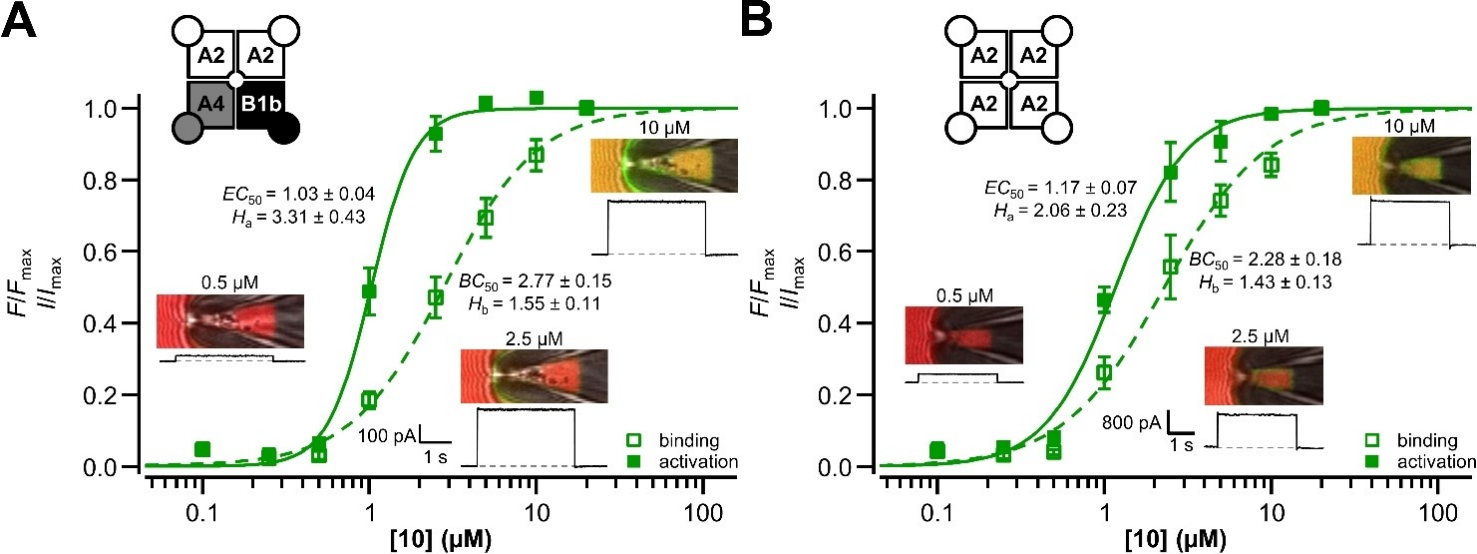
Ligand binding and activation in (A) hetero‐ and (B) homotetrameric CNG channels measured by cPCF. The obtained data are expressed as concentration‐activation and concentration‐binding relationships. Hill coefficients as well as *EC*_50_ and *BC*_50_ values are given next to the graphs. Fitting was accomplished with either Equation 1 or 2 (Experimental Section). The insets display simultaneously recorded current traces and confocal difference images, reflecting the portion of bound ligands, at various concentrations of **10**.

Herein, we determined the binding‐gating relation for the most potent fluorescent agonist **10** on homo‐ and heterotetrameric olfactory CNG channels using cPCF (Figure 4). As expected from the above data, the binding profile of **10** was similar for the two different types of ion channels, yielding similar *BC*_50_ and *H*_b_ values. The Hill coefficients are small (1.55 and 1.43), which is a common characteristic of such conjugates.^10^ The obtained *EC*_50_ values were smaller than the corresponding *BC*_50_ values. This shows that the channel is already maximally activated at submaximal liganding, which has been observed for CNG channels before.2a

### Binding modes of compounds 6, 9, 12 and 13 in HCN2 channels

**Figure 5:**
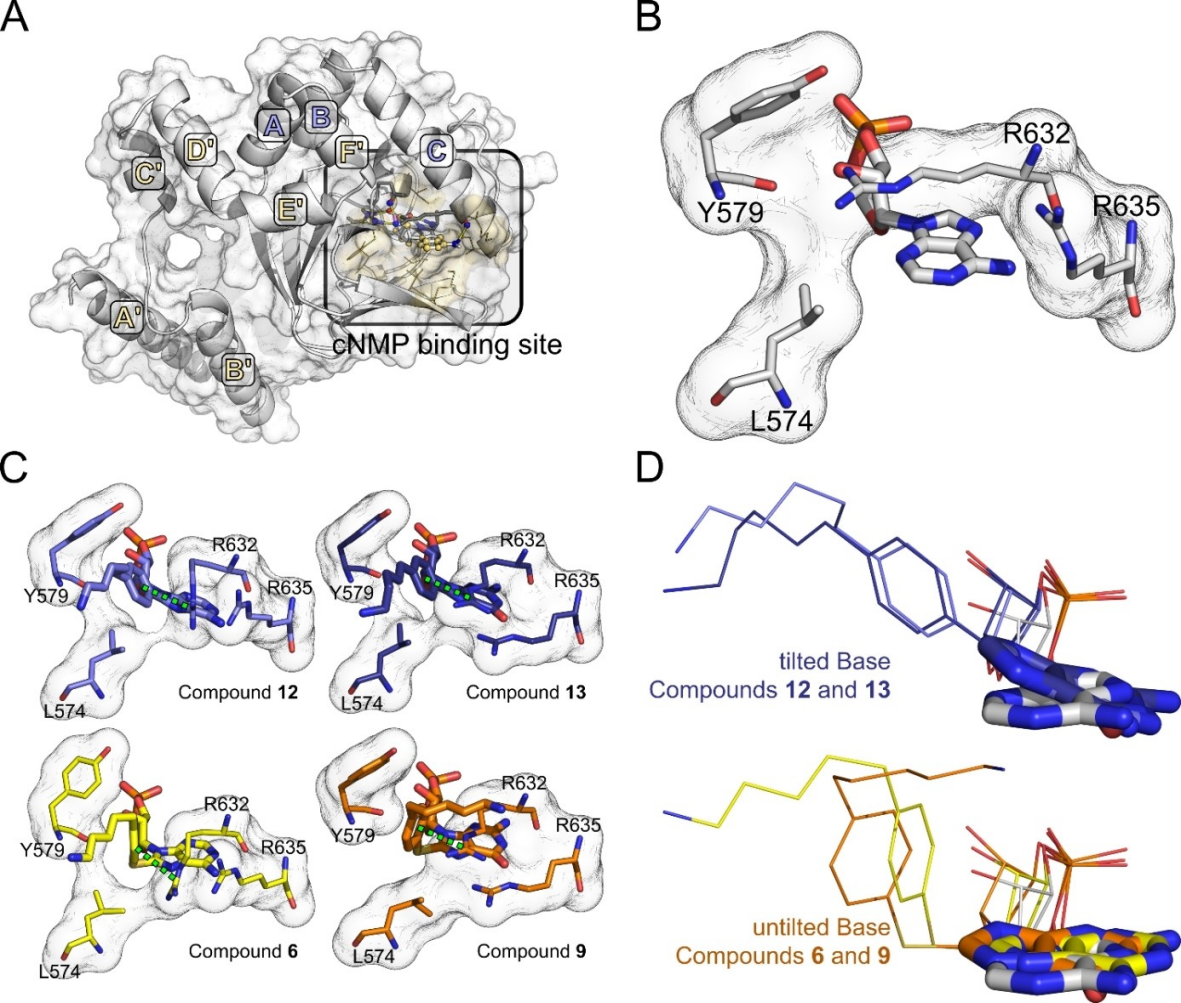
Differences in the crystallographically determined binding mode of cAMP and the predicted binding modes of cNMP derivatives **6**, **9**, **12** and **13**. (A) Overview of the systems used for structural analysis and induced fit docking (PDB ID: 1Q5O). The structure is depicted in white cartoon and surface representation. C‐linker helices are labeled in yellow, CNBD helices are labeled in blue. The cNMP binding site is highlighted with a black outline. (B) Crystallographically determined binding mode of cAMP in murine HCN2 (PDB ID: 1Q5O). Only those amino acids of HCN2 that change their rotameric state during induced fit docking of one of the derivatives are shown. The nucleotide is highlighted by thicker sticks. (C) Predicted binding modes of compounds **12** (blue), **13** (dark blue), **6** (yellow) and **9** (orange). The representation corresponds to the one used in B. Possible cation‐π interactions between R632 and the phenyl rings of the ligands are indicated by green dashed lines. (D) Superposition of the crystallographically determined binding mode of cAMP with the predicted binding modes of these compounds. The structure of the purine base is highlighted by thicker sticks. The colors correspond to those used in C.

To study how the binding modes of compounds **6**, **9**, **12**, and **13** differ from the binding mode of cAMP in HCN2 and to which extent conformational changes of the residues in the binding pocket are required to accommodate the bulky substituents of these compounds, we conducted Induced Fit Docking^13^ computations in the crystal structure of cAMP‐bound CL‐CNBD of HCN2J (PDB ID: 1Q5O,^14^ Figure 5A). For all compounds, the predicted geometry of the cyclic monophosphate corresponded to the geometry observed for cAMP in the crystal structure (RMSD: ≤0.75 Å, Figure 5B, C), and no side chain rotamers of the amino acids surrounding this ligand changed during the Induced Fit Docking process. This result shows that the docking algorithm is capable of reproducing the crystallographic pose of the activity‐determining structural element while maintaining the correct conformation of the binding pocket, which suggests that also the conformational changes of the binding pocket, necessary to accommodate the bulky substituents of compounds **6**, **9**, **12** and **13**, can be correctly predicted.

All compounds are bound exclusively in the *syn* conformation, as otherwise the aminoalkylphenyl or aminoalkylthiophenol substituent could not be accommodated (Figure 5C). In addition, the side chain rotamer of R632 changed in all cases, and the side chain rotamer of L574 in the case of compound **9**, to make space for the bulky phenyl ring (Figure 5C). The rotamer change of R632 allows for a cation‐π interaction with the phenyl ring, which may contribute to the stabilization of the binding pose (Figure 5C). In all compounds, the alkyl chain is predominantly stabilized by hydrophobic contacts with the surrounding residues (supplementary information), while the amino group extends towards the solvent, preventing its desolvation and the associated enthalpic penalty.

Notably, the aminoalkylphenyl‐substituted compounds **12** and **13** do not form a hydrogen bond with the backbone of R632, in contrast to the natural agonist cAMP, while in the complexes containing the aminoalkylthiophenol‐substituted compounds **6** and **9** this hydrogen bond is retained. This phenomenon can be attributed to the additional sulfur atom in compounds **6** and **9** that allows the phenyl ring to orient almost perpendicularly relative to the ring system of the base, which is not possible for compounds **12** and **13** where the sulfur atom is absent (Figure 5D). As a result, the ring system of the base in compounds **12** and **13** is tilted, with respect to the usual binding mode of cyclic nucleotides in HCN2 channels, to accommodate the bulky phenyl substituent (Figure 5D). This orientation likely leads to weaker interactions with the protein, which explains the different effects of the molecules on HCN2 channels described in Figure 3. Moreover, this finding supports the entire data from Table 1 due to the structural similarity of the CNBD of CNG and HCN channels.^15^

## Conclusion

In summary, we report the synthesis and functional characterization of two novel fluorophore‐labeled cyclic nucleotides. Both fluorescent nucleotide derivatives were prepared with a novel heterobifunctional cross‐linking reagent that can easily undergo nucleophilic substitutions as well as coupling reactions with active esters of fluorescent dyes after deprotection. The probes are potent agonists of hetero‐ and homotetrameric CNG as well as HCN2 channels with affinities in the nanomolar range. The high affinity of the dye conjugates towards the CNBD of the aforementioned ion channels is attributed to hydrogen bonds, similar to the natural ligand, as well as hydrophobic and cation‐π interactions. To enable an efficient interaction between the thiophenol‐substituted compound and the protein, the sulfur atom is mandatory.

Furthermore, our synthesis strategies allow us to prepare several novel phenyl‐ and thiophenol‐substituted cAMP and cGMP derivatives, which can be used as ligands for investigating cyclic nucleotide‐regulated ion channels regarding, for example, subtype specificity, subunit composition, or affinity of the CNBDs. In addition, the thiophenol‐substituted nucleotides are potential probes to investigate also related cAMP‐ and cGMP‐dependent protein kinases as well as exchange proteins directly activated by cAMP, because the previously reported compounds 8‐pCPT‐cAMP and 8‐pCPT‐cGMP have already shown useful properties for these target proteins.^16^

## Experimental Section

**General information**: Chemicals, including solvents and reagents, were purchased from commercial sources and used without further purification. Thin layer chromatography sheets (ALUGRAM SIL G/UV_254_) as well as silica gel for column chromatography (0.04–0.063 mm) were bought from Macherey‐Nagel (Düren, Germany) and utilized with suitable solvent systems.

Reversed‐phase high‐performance liquid chromatography (RP–HPLC) was performed on an Agilent Technologies 1100 Series system (Waldbronn, Germany) with appropriate solvent delivery pumps (G1361A), a dual loop autosampler (G2258A) and a multi‐wavelength detector (G1365B). Analytical RP‐HPLC was conducted on an AppliChrom (Oranienburg, Germany) OTU LipoMare C_18_ column (250×4.6 mm) with 5 μm particle size as stationary phase and a flow rate of 1 mL/min. Purification of the cyclic nucleotide derivatives was carried out on an AppliChrom OTU LipoMare C_18_ column (250×20 mm) at an appropriate flow rate of 15 mL/min and with 5 μm silica as stationary phase. Product fractions were isolated with an Agilent Technologies fraction collector (G1364C). The applied eluents were 25 mM (pH 7) triethylammonium acetate buffer (A) as well as acetonitrile (B). The used gradients were linear from 0 min (100 % A) to 25 min (75 % A) and from 0 min (100 % A) to 40 min (60 % A) for the dye conjugates. The substances were simultaneously detected either at 230 and 260 nm or at 260 and 560 nm for the dye conjugates.

^1^H and ^13^C NMR spectra were recorded at 300 K on a Bruker Avance I 300 MHz spectrometer (Karlsruhe, Germany). Chemical shifts are reported in parts per million, relative to the residual solvent signals of DMSO‐*d*_6_, AcOH‐*d*_4_ and D_2_O.^17^ Coupling constants (*J*) are given in hertz (Hz).

High‐resolution electrospray and atmospheric pressure chemical ionization mass spectrometry measurements were carried out on a Bruker Daltonics micrOTOF system (Bremen, Germany), equipped with an automatic syringe pump for sample injection. The standard electrospray ion source was used to generate ions and the instrument was calibrated in the *m*/*z* range 50 to 3000 utilizing an internal calibration standard (Tunemix solution) from Agilent Technologies.

### Chemical syntheses

*Synthesis of compound **2***: 1,4‐Dibromobenzene (**1**, 4.72 g, 20 mmol) was dissolved in 40 mL dry tetrahydrofuran and the solution was cooled to −78 °C under argon. *n*‐Butyllithium (2.5 M in hexanes, 8 mL, 20 mmol) was slowly added, and the reaction mixture was stirred for 30 min under argon. Subsequently, 1,5‐dibromopentane (13.8 g, 8.17 mL, 60 mmol) was added, and the mixture was stirred for further 2 h at −78 °C under argon. After warming to room temperature, the solution was diluted with 200 mL diethyl ether, washed twice with water and the organic layer was dried with magnesium sulfate. The solvent and the excess of 1,5‐dibromopentane were removed and the residue was purified by silica gel column chromatography using *n*‐hexane/ethyl acetate (20 : 1) as eluent, to yield the product as colorless oil (3.61 g, 11.8 mmol, 59 %). ^1^H NMR (300 MHz, DMSO‐*d*_6_): *δ*=7.45 (2H, d, *J*=8.4 Hz), 7.16 (2H, d, *J*=8.4 Hz), 3.50 (2H, t, *J*=6.7 Hz), 2.54 (2H, t, *J*=7.6 Hz), 1.87–1.74 (2H, m), 1.62–1.49 (2H, m), 1.43–1.31 (2H, m); ^13^C NMR (75 MHz, DMSO‐*d*_6_): *δ*=141.4, 131.0, 130.5, 118.6, 35.0, 34.2, 32.0, 29.7, 27.1; HRMS (APCI): *m*/*z* calcd for C_11_H_14_Br_2_ [*M*]^+^ 303.9457, found 303.9455.

*Synthesis of compound **3***: Alkyl bromide **2** (765.1 mg, 2.5 mmol) and 20 mL 7 M ammonia in methanol were heated at 100 °C for 1 h in a sealed reaction vessel with stirring. After that, the solution was cooled to ambient temperature and the solvent was evaporated under reduced pressure. The obtained residue was taken up in 20 mL dry tetrahydrofuran and triethylamine (506.0 mg, 693.1 μL, 5 mmol) was added. The suspension was added to di‐*tert*‐butyl dicarbonate (545.6 mg, 2.5 mmol), and the reaction mixture was stirred for 2 h at 50 °C under argon. Afterwards, undissolved salts were filtered off, washed with tetrahydrofuran and the solvent was removed under vacuum. Purification of the residue by column chromatography on silica gel with *n*‐hexane/ethyl acetate (3 : 1) as eluent yielded the product as colorless oil (710.2 mg, 2.08 mmol, 83 %). ^1^H NMR (300 MHz, DMSO‐*d*_6_): *δ*=7.44 (2H, d, *J*=8.3 Hz), 7.15 (2H, d, *J*=8.3 Hz), 6.75 (1H, t, *J*=5.4 Hz), 2.95–2.80 (2H, m), 2.52 (2H, t, *J*=7.6 Hz), 1.59–1.46 (2H, m), 1.43–1.31 (2H, m), 1.35 (9H, s), 1.28–1.15 (2H, m); ^13^C NMR (75 MHz, DMSO‐*d*_6_): *δ*=155.6, 141.6, 131.0, 130.5, 118.6, 77.3, 39.7, 34.4, 30.3, 29.2, 28.2, 25.7; HRMS (ESI): *m*/*z* calcd for C_16_H_24_BrNNaO_2_ [*M*+Na]^+^ 364.0883, found 364.0873.

*Synthesis of compound **4***: The protected aryl bromide **3** (342.3 mg, 1 mmol) was dissolved in 8 mL freshly degassed dry 1,4‐dioxane and added to tris(dibenzylideneacetone)dipalladium(0) (45.8 mg, 50 μmol), 4,5‐bis(diphenylphosphino)‐9,9‐dimethylxanthene (57.9 mg, 100 μmol) and potassium thioacetate (137.1 mg, 1.2 mmol) under argon. The reaction mixture was stirred for 24 h at 80 °C under argon. After cooling to room temperature, the mixture was precipitated in 100 mL *n*‐hexane, filtered and the solvent was removed. The obtained residue was purified by silica gel column chromatography using *n*‐hexane/ethyl acetate (3 : 1) as eluent, to afford the product as colorless solid (242.9 mg, 720 μmol, 72 %). ^1^H NMR (300 MHz, DMSO‐*d*_6_): *δ*=7.31 (2H, d, *J*=8.5 Hz), 7.27 (2H, d, *J*=8.5 Hz), 6.77 (1H, t, *J*=5.5 Hz), 2.94–2.85 (2H, m), 2.59 (2H, t, *J*=7.6 Hz), 2.40 (3H, s), 1.62–1.50 (2H, m), 1.44–1.34 (2H, m), 1.36 (9H, s), 1.31–1.19 (2H, m); ^13^C NMR (75 MHz, DMSO‐*d*_6_): *δ*=193.8, 155.5, 144.0, 134.2, 129.3, 124.5, 77.2, 39.7, 34.7, 30.3, 30.0, 29.2, 28.2, 25.9; HRMS (ESI): *m*/*z* calcd for C_18_H_27_NNaO_3_S [*M*+Na]^+^ 360.1604, found 360.1604.

*Synthesis of compound **6***: The brominated cyclic nucleotide **5** (43.0 mg, 100 μmol) was dissolved in 3 mL of a 0.1 M sodium hydroxide solution and heated to 70 °C. Subsequently, **4** (50.6 mg, 150 μmol) dissolved in 3 mL 1,4‐dioxane was slowly added, and the obtained solution was stirred for 4 h at 70 °C under argon. After that, 2 mL 2 M hydrochloric acid were added, and the mixture was stirred for further 2 h at 70 °C. The volume was reduced to a minimum and the residue was taken up in 25 mM triethylammonium acetate buffer (pH 7) and purified by RP‐HPLC. The solvent of the isolated fractions was removed under reduced pressure and the obtained solid was further desiccated in a high vacuum, to yield the product as colorless solid (35.5 mg, 68 μmol, 68 %). ^1^H NMR (300 MHz, 1 M DCl in D_2_O): *δ*=7.92 (1H, s), 7.00 (2H, d, *J*=8.0 Hz), 6.82 (2H, d, *J*=8.0 Hz), 5.88 (1H, s), 4.77–4.71 (1H, m), 4.43 (1H, d, *J*=5.4 Hz), 4.13–3.85 (2H, m), 3.82–3.65 (1H, m), 2.55 (2H, t, *J*=7.5 Hz), 2.16 (2H, t, *J*=7.4 Hz), 1.35–1.09 (4H, m), 1.04–0.89 (2H, m); ^13^C NMR (75 MHz, 1 M DCl in D_2_O): *δ*=153.2, 149.4, 147.8, 145.6, 143.6, 134.2, 130.0, 123.0, 118.6, 92.6, 76.7 (d, *J*=3.6 Hz), 71.9 (d, *J*=4.5 Hz), 71.5 (d, *J*=8.1 Hz), 67.3 (d, *J*=6.7 Hz), 39.3, 34.4, 29.7, 26.4, 25.1; HRMS (ESI): *m*/*z* calcd for C_21_H_28_N_6_O_6_PS [*M*+H]^+^ 523.1523, found 523.1516.

*Synthesis of compound **9***: This cyclic nucleotide derivative was prepared according to the synthesis of **6** with **8** (44.6 mg, 100 μmol), to afford the product as colorless solid (28.5 mg, 53 μmol, 53 %). ^1^H NMR (300 MHz, 2 M DCl in D_2_O): *δ*=6.29 (2H, d, *J*=8.1 Hz), 6.04 (2H, d, *J*=8.1 Hz), 4.91 (1H, s), 3.99–3.87 (1H, m), 3.70 (1H, d, *J*=5.6 Hz), 3.30 (1H, ddd, *J*=21.5, 9.1, 4.5 Hz), 3.18–3.07 (1H, m), 3.05–2.92 (1H, m), 1.72 (2H, t, *J*=7.5 Hz), 1.33 (2H, t, *J*=7.5 Hz), 0.48‐0.24 (4H, m), 0.16–0.05 (2H, m); HRMS (ESI): *m*/*z* calcd for C_21_H_27_N_6_NaO_7_PS [*M*+Na]^+^ 561.1292, found 561.1283.

*Synthesis of compound **7***: Compound **6** (3.16 mg, 6.05 μmol) was suspended in 1 mL dry *N*,*N*‐dimethylformamide and triethylamine (11.13 mg, 15.25 μL, 110 μmol) was added. Afterwards, the *N*‐hydroxysuccinimide ester of Cy3B (3.62 mg, 5.50 μmol) was added, and the reaction mixture was stirred under argon overnight at room temperature. Subsequently, the volume of the mixture was reduced to a minimum and the residue was taken up in 25 mM triethylammonium acetate buffer (pH 7) and purified by RP‐HPLC. The solvent of the collected fractions was removed under reduced pressure and the obtained solid was further dried in a high vacuum, to yield the product as red solid (2.58 mg, 2.42 μmol, 44 %). RP‐HPLC: *t*_R_=39.1 min; HRMS (ESI): *m*/*z* calcd for C_52_H_56_N_8_O_11_PS_2_ [*M*−H]^−^ 1063.3253, found 1063.3251.

*Synthesis of compound **10***: This fluorophore‐labeled cyclic nucleotide was synthesized according to the preparation of **7** with **9** (3.26 mg, 6.05 μmol), to afford the product as red solid (3.09 mg, 2.86 μmol, 52 %). RP‐HPLC: *t*_R_=38.1 min; HRMS (ESI): *m*/*z* calcd for C_52_H_56_N_8_O_12_PS_2_ [*M*−H]^−^ 1079.3202, found 1079.3152.

*Synthesis of compound **11***: The protected aryl bromide **3** (342.3 mg, 1 mmol) was dissolved in 10 mL freshly degassed 1,4‐dioxane and added to [1,1′‐bis(diphenylphosphino)ferrocene]palladium(II) dichloride (109.8 mg, 150 μmol), bis(pinacolato)diboron (330.1 mg, 1.3 mmol) and potassium acetate (294.4 mg, 3 mmol) under argon. The reaction mixture was stirred for 4 h at 80 °C under argon. After cooling to room temperature, the mixture was precipitated in 100 mL *n*‐hexane, filtered and the solvent was removed. The obtained residue was purified by silica gel column chromatography using *n*‐hexane/ethyl acetate (3 : 1) as eluent, yielding the product as colorless oil (334.8 mg, 860 μmol, 86 %). ^1^H NMR (300 MHz, DMSO‐*d*_6_): *δ*=7.58 (2H, d, *J*=7.9 Hz), 7.19 (2H, d, *J*=7.9 Hz), 6.74 (1H, t, *J*=5.5 Hz), 2.94–2.81 (2H, m), 2.56 (2H, t, *J*=7.5 Hz), 1.61–1.47 (2H, m), 1.43–1.32 (2H, m), 1.35 (9H, s), 1.30–1.18 (2H, m), 1.27 (12H, s); ^13^C NMR (75 MHz, DMSO‐*d*_6_): *δ*=155.5, 145.8, 134.5, 127.8, 125.6, 83.4, 77.2, 39.7, 35.2, 30.4, 29.2, 28.2, 25.8, 24.6; HRMS (ESI): *m*/*z* calcd for C_22_H_36_BNNaO_4_ [*M*+Na]^+^ 412.2630, found 412.2638.

*Synthesis of compound **12***: Boronic ester **11** (58.4 mg, 150 μmol) dissolved in 4 mL freshly degassed 1,4‐dioxane and the brominated cyclic nucleotide **5** (43.0 mg, 100 μmol) as well as potassium phosphate (63.7 mg, 300 μmol) dissolved in 4 mL freshly degassed water were added in turn to chloro(sodium‐2‐dicyclohexylphosphino‐2′,6′‐dimethoxy‐1,1′‐biphenyl‐3′‐sulfonate)[2‐(2′‐amino‐1,1′‐biphenyl)]palladium(II) (8.2 mg, 10 μmol) under argon. The reaction mixture was stirred for 4 h at 70 °C under argon. Afterwards, 2 mL 2 M hydrochloric acid were added, and the mixture was stirred for further 2 h at 70 °C. After that, the volume was reduced to a minimum and the residue was taken up in 25 mM triethylammonium acetate buffer (pH 7) and purified by RP‐HPLC. The solvent of the collected fractions was removed under reduced pressure and the obtained solid was further dried in a high vacuum, to afford the product as colorless solid (31.4 mg, 64 μmol, 64 %). ^1^H NMR (300 MHz, AcOH‐*d*_4_): *δ*=8.48 (1H, s), 7.66 (2H, d, *J*=8.1 Hz), 7.42 (2H, d, *J*=8.1 Hz), 5.97 (1H, s), 5.38–5.23 (1H, m), 4.92 (1H, d, *J*=5.5 Hz), 4.54–4.30 (2H, m), 4.28–4.13 (1H, m), 3.11 (2H, t, *J*=7.4 Hz), 2.76 (2H, t, *J*=6.5 Hz), 1.91–1.65 (4H, m), 1.59–1.41 (2H, m); ^13^C NMR (75 MHz, AcOH‐*d*_4_): *δ*=155.1, 153.3, 150.7, 148.7, 147.6, 131.1, 130.1, 125.5, 118.9, 94.0, 78.3, 73.2, 72.8, 68.4, 40.9, 36.2, 31.3, 27.9, 26.7; HRMS (ESI): *m*/*z* calcd for C_21_H_28_N_6_O_6_P [*M*+H]^+^ 491.1802, found 491.1781.

*Synthesis of compound **13***: This cyclic nucleotide derivative was prepared according to the synthesis of **12** with **8** (44.6 mg, 100 μmol), to yield the product as colorless solid (3.55 mg, 7 μmol, 7 %). RP‐HPLC: *t*_R_=21.0 min; HRMS (ESI): *m*/*z* calcd for C_21_H_28_N_6_O_7_P [*M*+H]^+^ 507.1752, found 507.1735.

**Molecular biology and heterologous expression of CNG and HCN channels**: The subunits CNGA2 (accession No. AF126808), CNGA4 (accession no. U12623) and CNGB1b (accession no. AF068572) of rat olfactory channels as well as mouse HCN2 channels (NM008226) were subcloned in front of the T7 promoter of pGEMHEnew. The corresponding cRNAs were produced with the mMESSAGE mMACHINE T7 Kit (Ambion, Austin, TX, USA).

Oocytes of *Xenopus laevis* were either purchased from Ecocyte® (Castrop‐Rauxel, Germany) or obtained surgically from female adults under anesthesia (0.3 % 3‐aminobenzoic acid ethyl ester). The procedures had approval from the authorized animal ethical committee of the Friedrich Schiller University Jena, and the methods were carried out according to the approved guidelines.

The oocytes were incubated for 105 min in Ca^2+^‐free Barth's solution containing collagenase A (3 mg/mL; Roche, Grenzach‐Wyhlen, Germany) and (in mM) 82.5 NaCl, 2 KCl, 1 MgCl_2_, 5 HEPES, pH 7.4. Oocytes at stages IV and V were injected with 50–130 ng cRNA encoding either CNGA2, CNGA2 : CNGA4 : CNGB1b (2 : 1 : 1 ratio) or HCN2 channels either manually or mediated by an injection robot (RoboInject®). The injected oocytes were incubated at 18 °C for up to 6 days in Barth's medium containing (in mM) 84 NaCl, 1 KCl, 2.4 NaHCO_3_, 0.82 MgSO_4_, 0.41 CaCl_2_, 0.33 Ca(NO_3_)_2_, 7.5 TRIS, cefuroxime (4.0 μg/mL) and penicillin/streptomycin (100 μg/mL), pH 7.4.

**Electrophysiology**: Macroscopic currents were recorded in inside‐out patches of the oocytes expressing hundreds to several thousand of the desired channels by using the patch‐clamp technique. The patch pipettes were pulled from quartz tubing (P‐2000, Sutter Instrument, Novato, USA) with an outer and inner diameter of 1.0 and 0.7 mm (VITROCOM, New Jersey, USA). The corresponding pipette resistance was 0.9–2.3 MΩ. The bath and pipette solution contained (in mM): 150 KCl, 1 EGTA, 20 HEPES (pH 7.4) for CNG channel measurements. For HCN channel measurements the bath solution contained (in mM): 100 KCl, 10 EGTA, 10 HEPES (pH 7.2) and the pipette (in mM) 120 KCl, 10 HEPES, 1 CaCl_2_ (pH 7.2). All experiments were performed at room temperature by using an Axopatch 200B amplifier (Axon Instruments, Foster City, CA, USA). Electrophysiology was controlled by the Patchmaster‐software (HEKA Elektronik Dr. Schulze GmbH, Lambrecht, Germany). The sampling rate was 5 kHz and the filter implemented in the amplifier (4‐pole Bessel) was set to 2 kHz. Measurements in HCN2 channels were started 3.5 minutes after patch excision, to minimize run down phenomena.^18^ The solutions with the different ligand concentrations to be studied were applied by a multi‐barrel device to the patches with a flow rate of 0.8 to 1.2 mL/min. The concentration of the fluorescent ligands was verified by UV/Vis spectroscopy.

**Confocal patch‐clamp fluorometry**: Ionic current and binding of the fluorescent ligand in macropatches were simultaneously measured by cPCF as described before.2a, 2b The patch pipettes were pulled from borosilicate glass tubing with an outer and inner diameter of 2.0 and 1.0 mm (Hilgenberg GmbH, Malsfeld, Germany). After fire polishing, the corresponding pipette resistance was 0.7–1.2 MΩ. The bath and pipette solution contained (in mM): 150 KCl, 1 EGTA, 5 HEPES (pH 7.4) for CNG channel measurements. To distinguish the fluorescence of the unbound fluorescent ligand from that of the bound fluorescent ligands, the fluorescent dye DY647 was added to the bath solution at a concentration of 5 μM. The fluorescent ligand and DY647 were excited at 543 and 633 nm, respectively, with a HeNe laser system and an AchroGate beam splitter. Fluorescence intensity from the bath and pipette solution were normalized on each other and the surplus of the green fluorescence of the patch dome with respect to the red fluorescence was used to quantify the bound ligands. The actual relative fluorescence (*F*) was normalized in each patch with respect to the maximum relative fluorescence (*F*_max_) at a saturating concentration of the fluorescent ligand.

**Fitting steady‐state concentration‐activation and concentration‐binding relationships**: Concentration‐activation relationships were fitted with the Igor software® to the data points by Equation (1):

(1) $$I/I{}_{\max}=1/\left(1+\left(EC{}_{50}/\left[\mathrm{nucleotide}\right]\right){}^{Ha}\right)$$

where *I* is the actual current amplitude and *I*_max_ the maximum current amplitude at saturating concentration of each cyclic nucleotide. *EC*_50_ is the ligand concentration generating half maximum current and *H*_a_ the respective Hill coefficient.

According to that, concentration‐binding relationships were fitted with the Igor software® by Equation (2):

(2) $$F/F{}_{\max}=1/\left(1+\left(BC{}_{50}/\left[\mathrm{nucleotide}\right]\right){}^{Hb}\right)$$

where *F* is the actual relative fluorescence intensity and *F*_max_ the maximum relative fluorescence intensity at saturating concentration of the fluorescent ligand. *BC*_50_ is the ligand concentration generating half maximum binding and *H*_b_ the Hill coefficient. All errors are given as mean ± S.E.M.

**Molecular docking of compounds 6, 9, 12 and 13 to murine HCN2**: In order to determine binding modes for compounds **6**, **9**, **12** and **13**, the Induced Fit Docking protocol^13^ implemented in the Schrödinger suite of programs was employed (Schrödinger Release 2018‐1: Induced Fit Docking protocol; Glide, Schrödinger, LLC, New York, NY, 2016; Prime, Schrödinger, LLC, New York, NY, 2018). First, the crystal structure of the cAMP‐bound CL‐CNBD of HCN2J (PDB ID: 1Q5O^14^) was prepared using the Protein Preparation Wizard^19^ (Schrödinger Release 2018‐1: Protein Preparation Wizard; Epik, Schrödinger, LLC, New York, NY, 2016; Impact, Schrödinger, LLC, New York, NY, 2016; Prime, Schrödinger, LLC, New York, NY, 2018) in the Maestro graphical user interface (GUI) of the Schrödinger suite (Schrödinger Release 2018‐1: Maestro, Schrödinger, LLC, New York, NY, 2018) by assigning bond orders, adding missing hydrogen atoms, converting selenomethionines to methionines and adding the missing side chains for residues D443, R447, E451, Q455, K510, I550, K552, K553, K567, N569, E571, K638, K639, S641, and I642. To assign protonation states to aspartate, glutamate, histidine, and lysine, tautomeric states to histidine and flip states of asparagine, glutamine, and histidine residues according to pH 7.4, the implementation of PROPKA^20^ in the Protein Preparation Wizard was used. Then the hydrogen atoms were energy‐minimized and all water molecules were removed from the complex. Subsequently, three‐dimensional structures for compounds **6**, **9**, **12** and **13** were generated in the Maestro GUI and further prepared using the LigPrep module (Schrödinger Release 2018‐1: LigPrep, Schrödinger, LLC, New York, NY, 2018) with default settings. To account for the greater spatial extent of these compounds compared to cAMP, the size of the outer box was adapted to allow docking of ligands with a length of ∼20 Å. In addition, the radius around the ligand pose was increased in which residues are selected for refinement with Prime from 5.0 Å to 8.0 Å. For each ligand, the complex with the lowest IFDScore^13^ was selected for further refinement with the Minimization Monte Carlo algorithm implemented in Prime (default settings).

## Conflict of interest

The authors declare no conflict of interest.

## Supporting information

As a service to our authors and readers, this journal provides supporting information supplied by the authors. Such materials are peer reviewed and may be re‐organized for online delivery, but are not copy‐edited or typeset. Technical support issues arising from supporting information (other than missing files) should be addressed to the authors.

Supplementary
