# Supplementary material for: Fluorophore‐Labeled Cyclic Nucleotides as Potent Agonists of Cyclic Nucleotide‐Regulated Ion Channels
Source: Chembiochem. 2020 May 4;21(16):2311–20. doi: 10.1002/cbic.202000116 (PMC7497086; doi:10.1002/cbic.202000116)
Supplement: Supplementary file 1 — Supplementary [file CBIC-21-2311-s001.pdf]

ChemBioChem

Supporting Information

Fluorophore-Labeled Cyclic Nucleotides as Potent Agonists of Cyclic Nucleotide-Regulated Ion Channels

Marco Lelle, Maik Otte, Michele Bonus, Holger Gohlke, and Klaus Benndorf*© 2020 The Authors. Published by Wiley-VCH Verlag GmbH & Co. KGaA. This is an open access article under the terms of the Creative Commons Attribution License, which permits use, distribution and reproduction in any medium, provided the original work is properly cited.

Synthesized compounds:

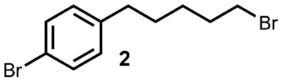

2

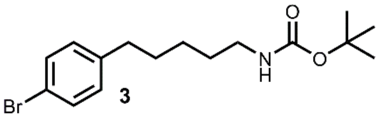

3

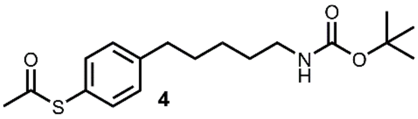

4

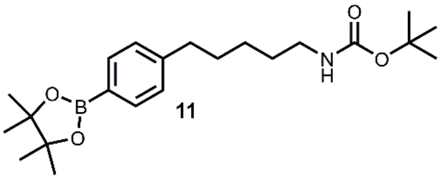

11

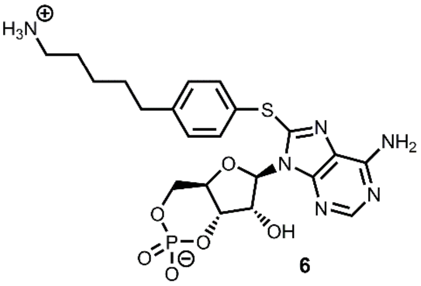

6

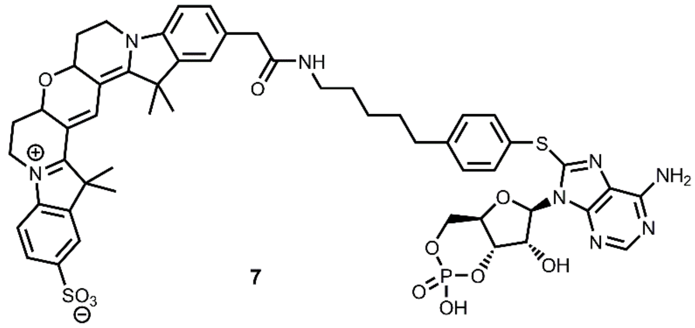

7

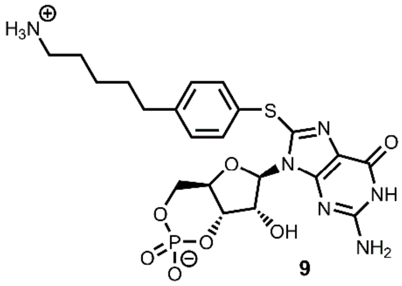

9

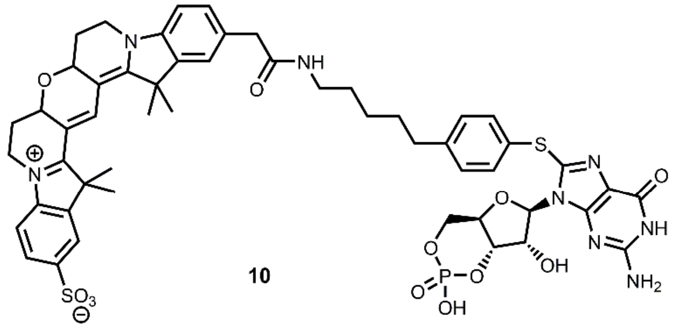

10

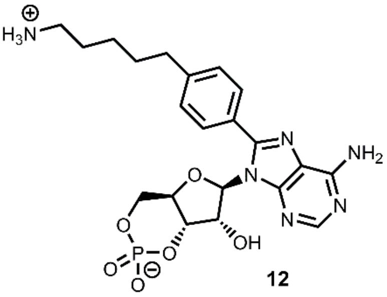

12

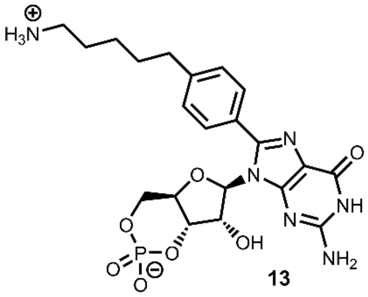

13

^{1}H NMR spectrum of compound **2** recorded in DMSO-d_6 at 300 K (300 MHz):

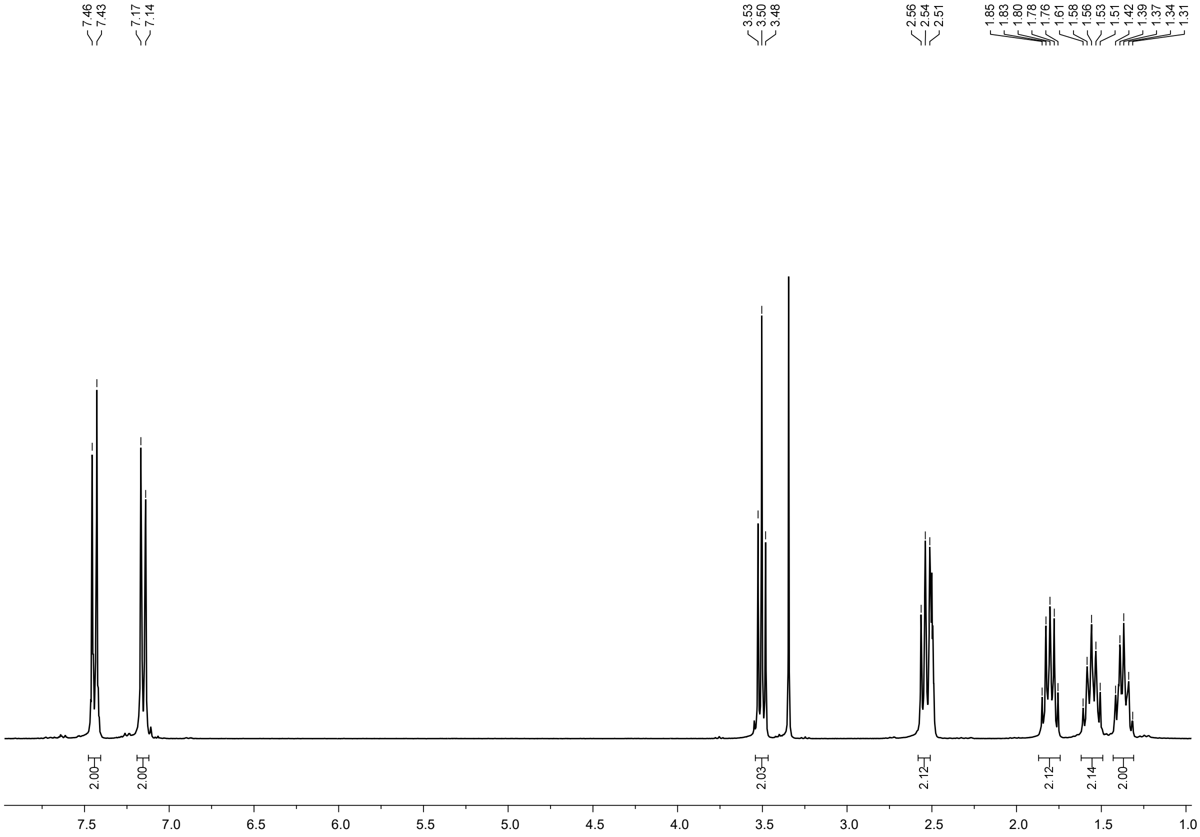

^{13}C NMR spectrum of compound **2** recorded in DMSO-d_6 at 300 K (75 MHz):

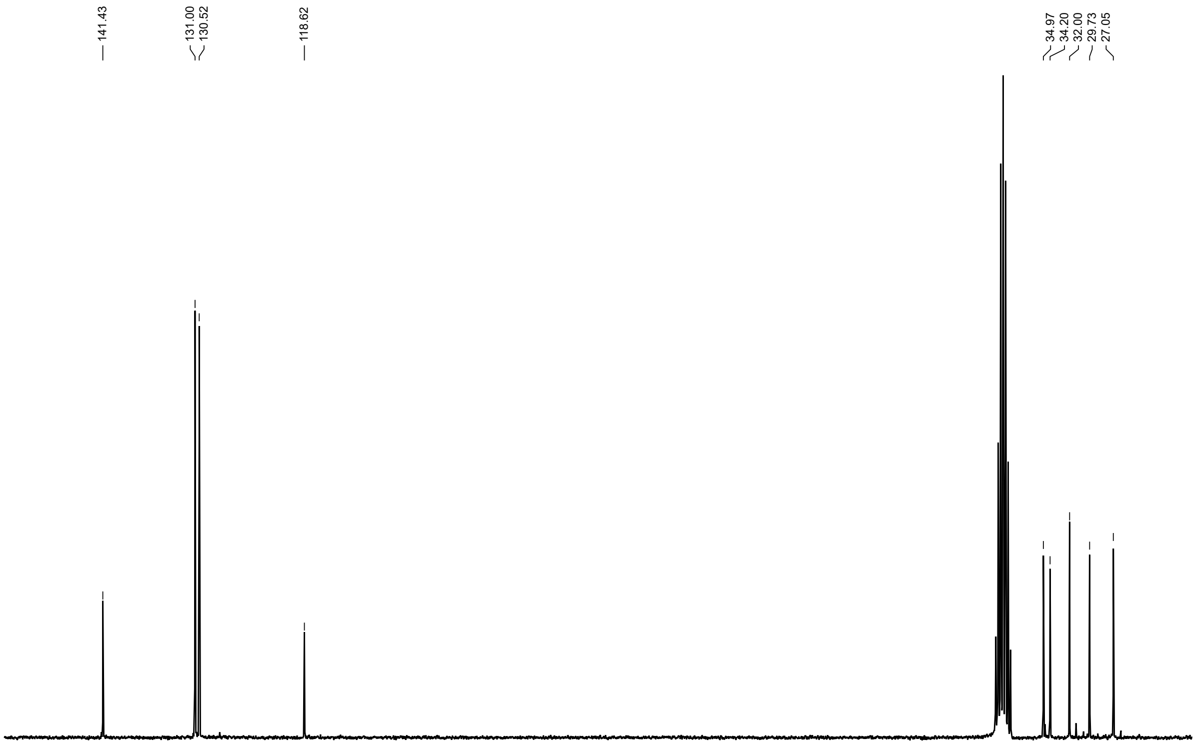

^1H NMR spectrum of compound **3** recorded in DMSO-d_6 at 300 K (300 MHz):

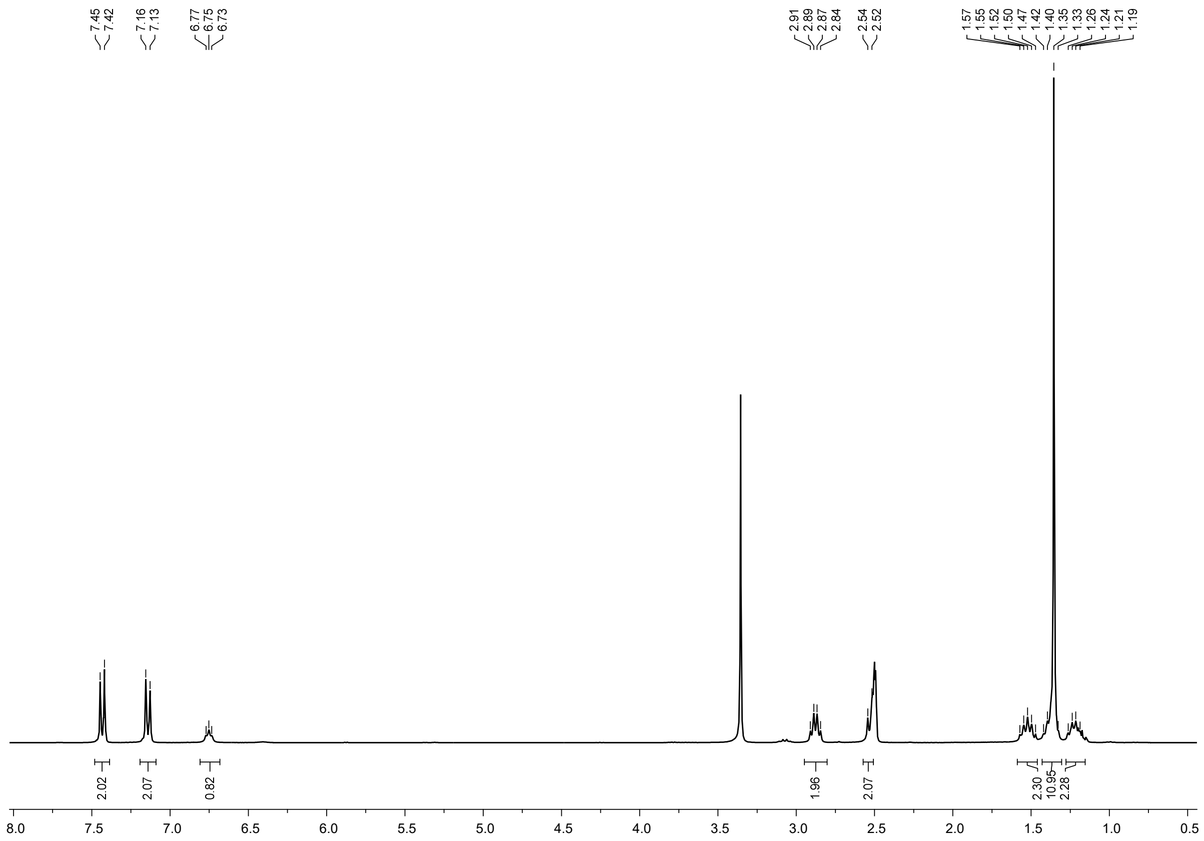

^{13}C NMR spectrum of compound **3** recorded in DMSO-d_6 at 300 K (75 MHz):

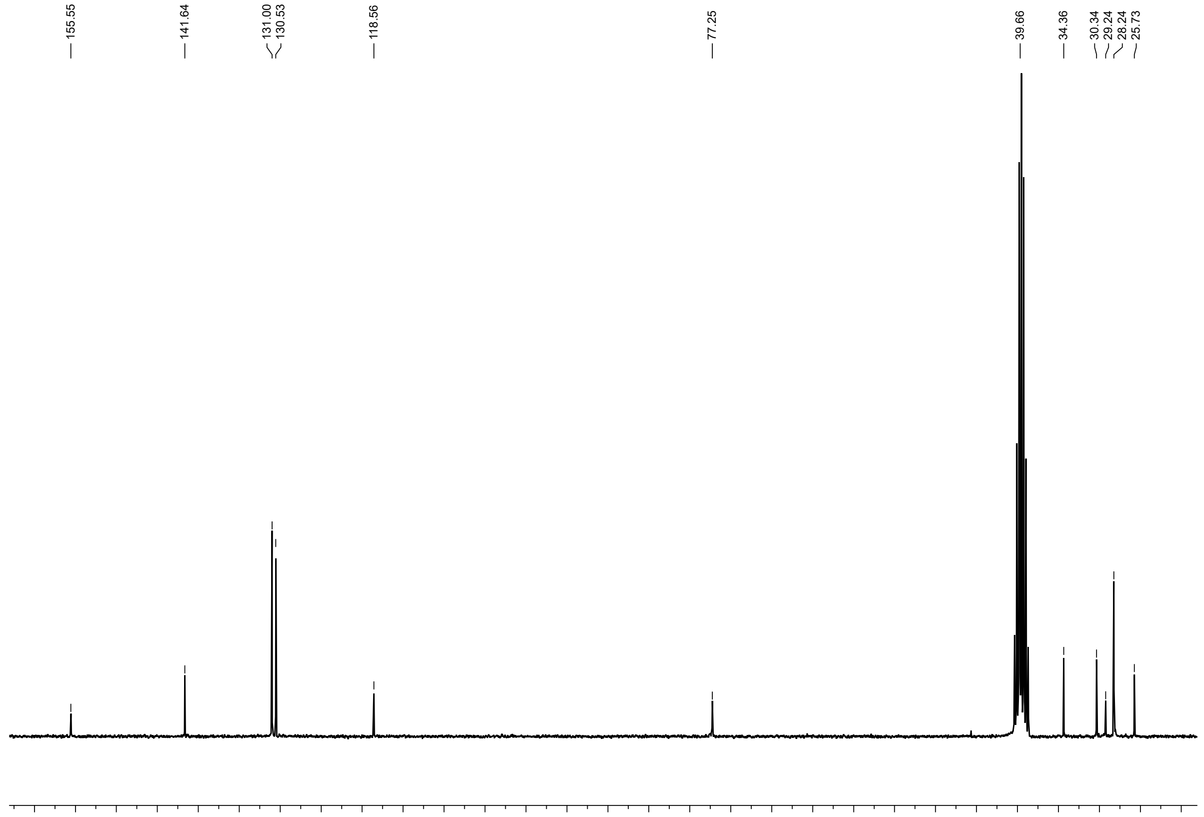

^{1}H NMR spectrum of compound **4** recorded in DMSO-d$_6$ at 300 K (300 MHz):

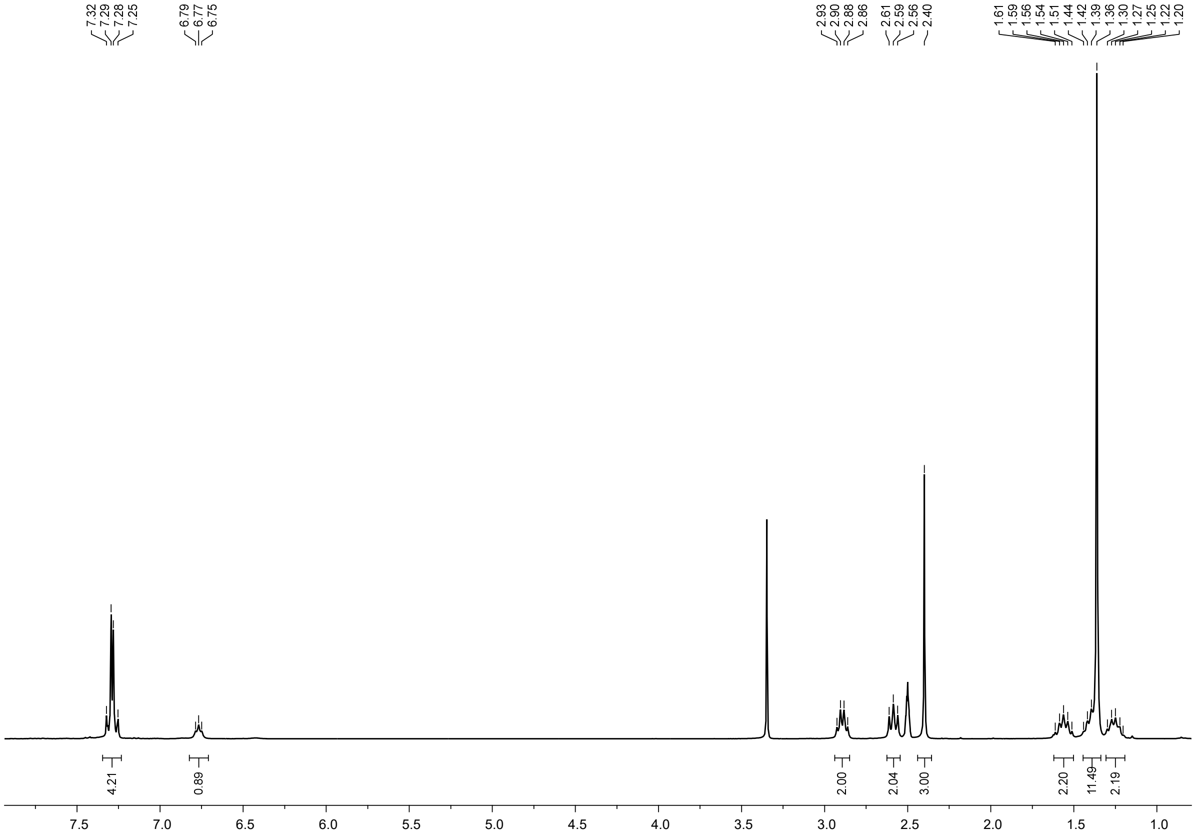

^{13}C NMR spectrum of compound **4** recorded in DMSO-d$_6$ at 300 K (75 MHz):

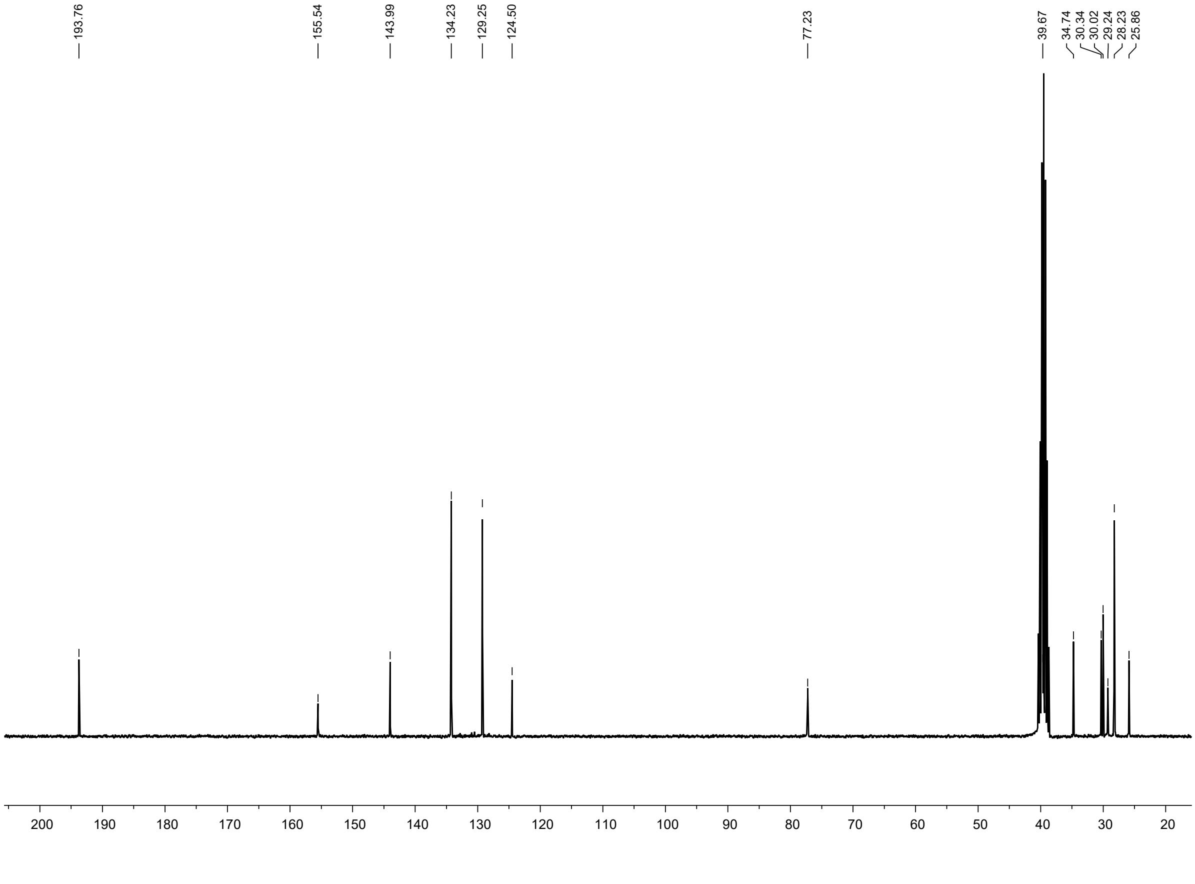

^{1}H NMR spectrum of compound **6** recorded in 1 M DCl in D_2O at 300 K (300 MHz):

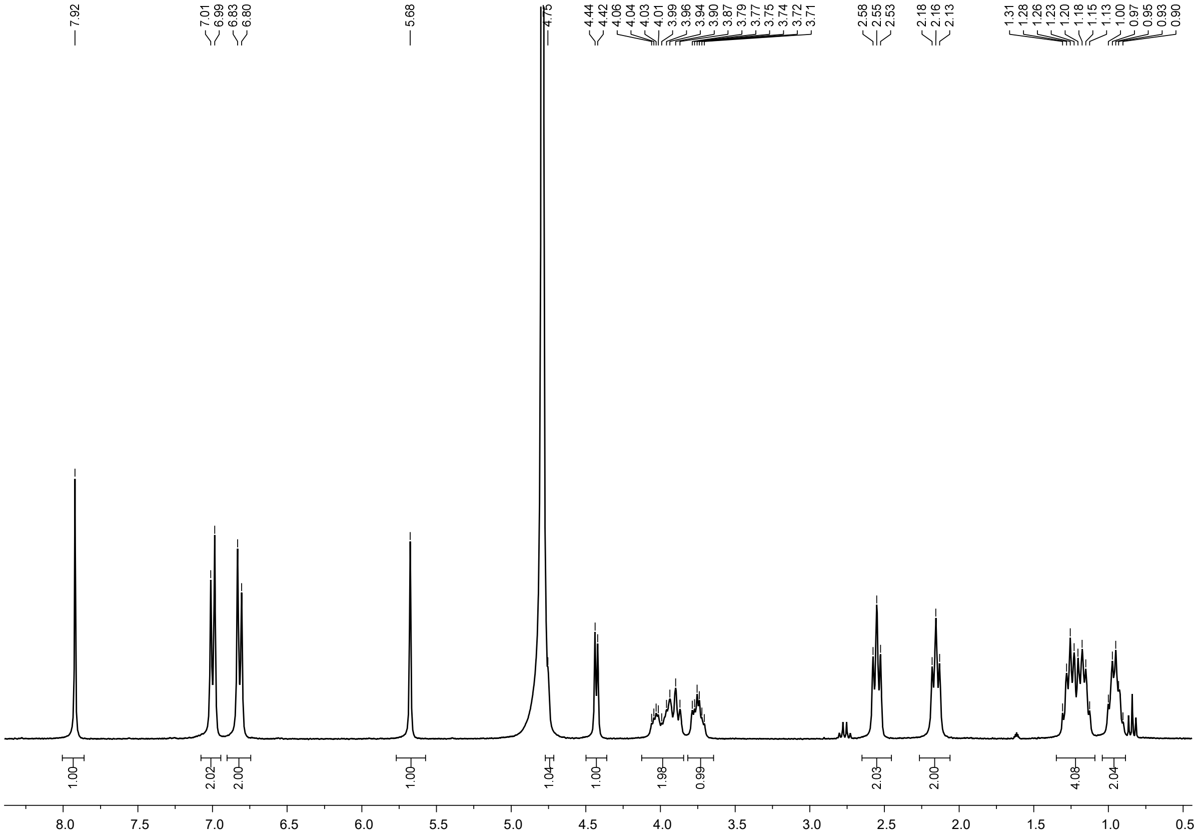

^{13}C NMR spectrum of compound **6** recorded in 1 M DCl in D_2O at 300 K (75 MHz):

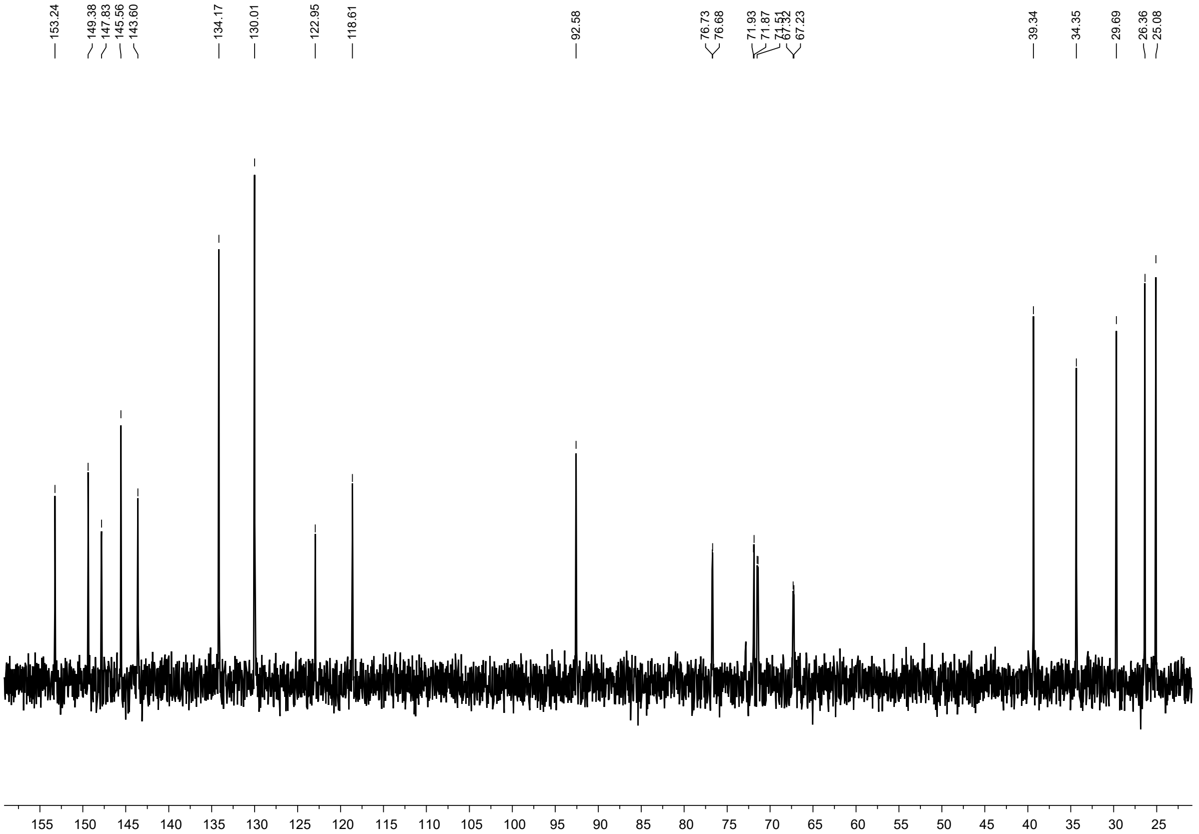

^{1}H NMR spectrum of compound **9** recorded in 2 M DCl in D_2O at 300 K (300 MHz):

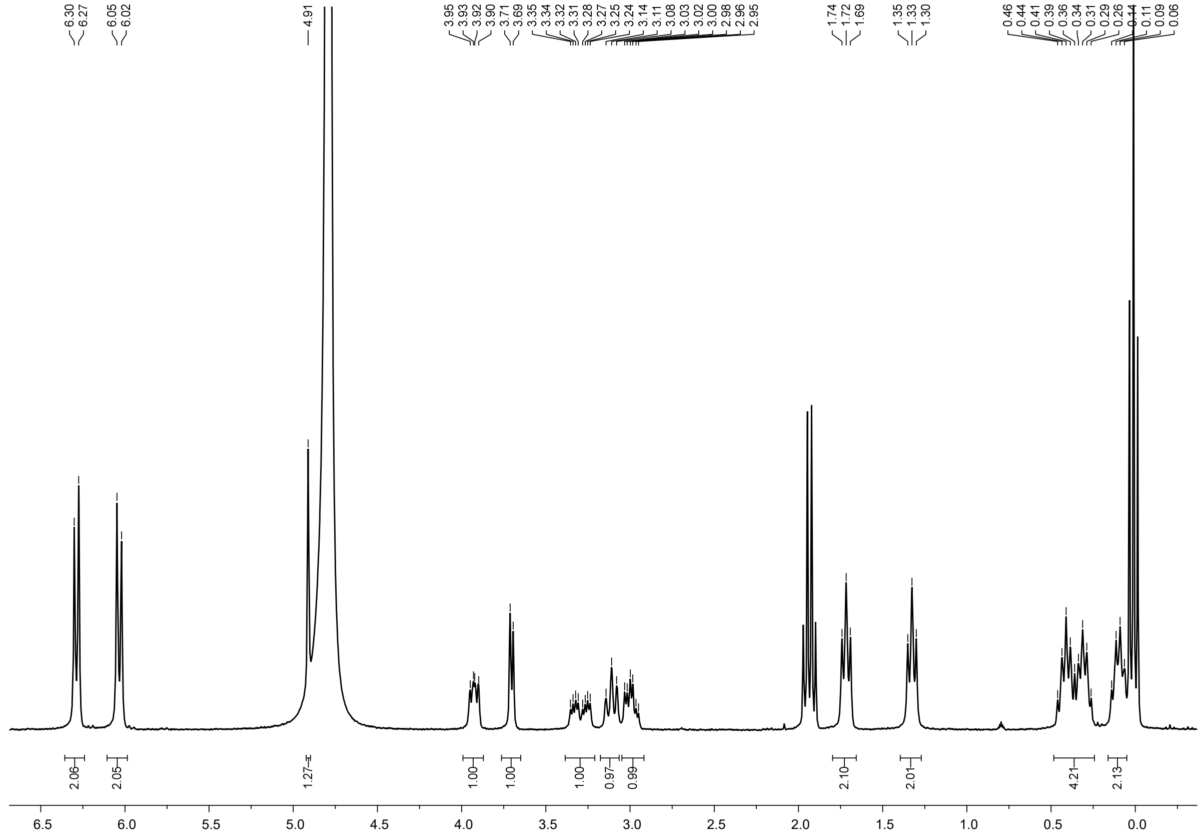

^{1}H NMR spectrum of compound **11** recorded in DMSO-d_6 at 300 K (300 MHz):

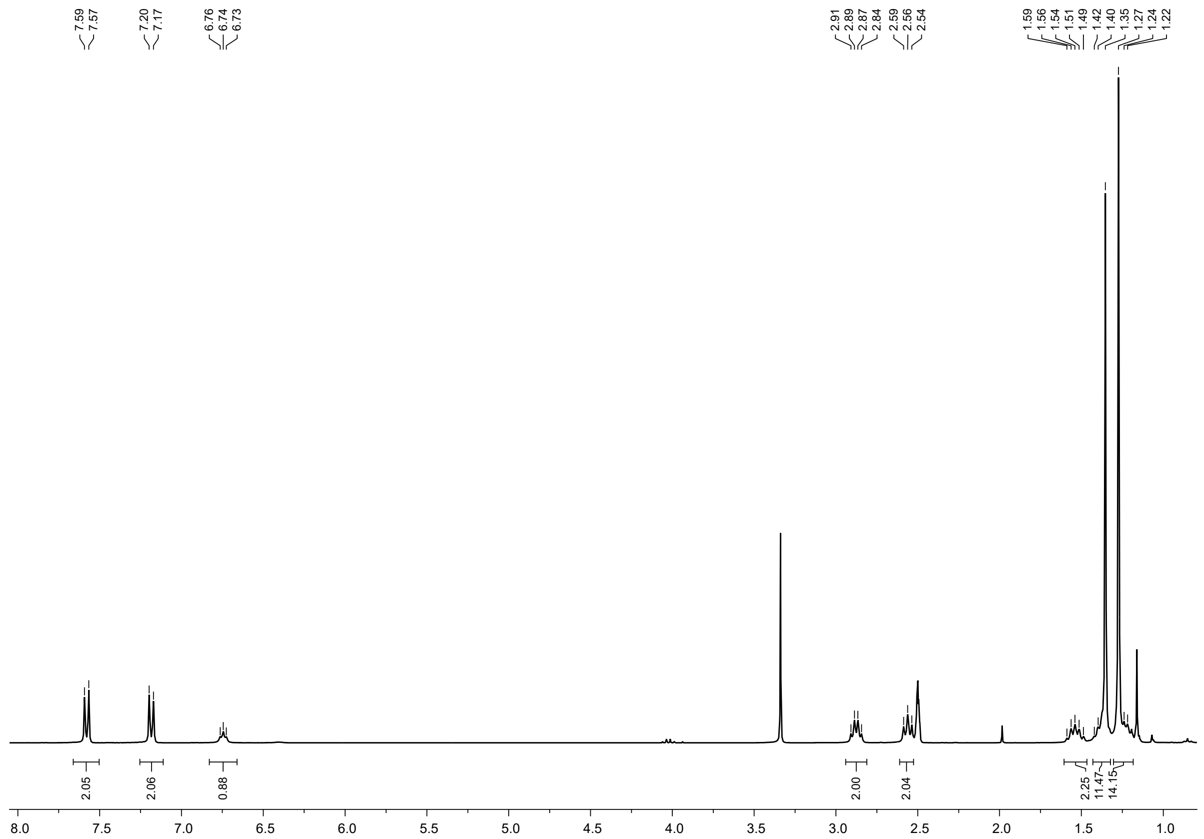

^{13}C NMR spectrum of compound **11** recorded in DMSO-d_6 at 300 K (75 MHz):

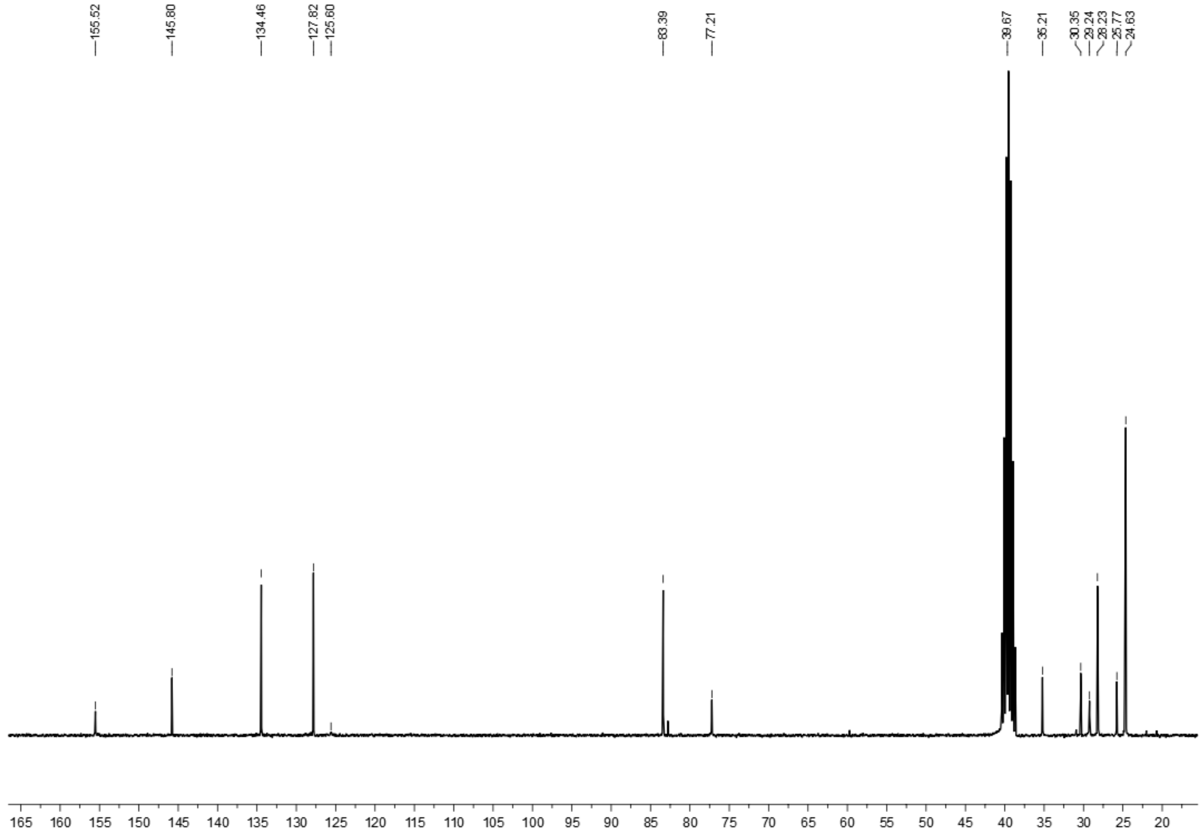

^{1}H NMR spectrum of compound **12** recorded in AcOH-d_4 at 300 K (300 MHz):

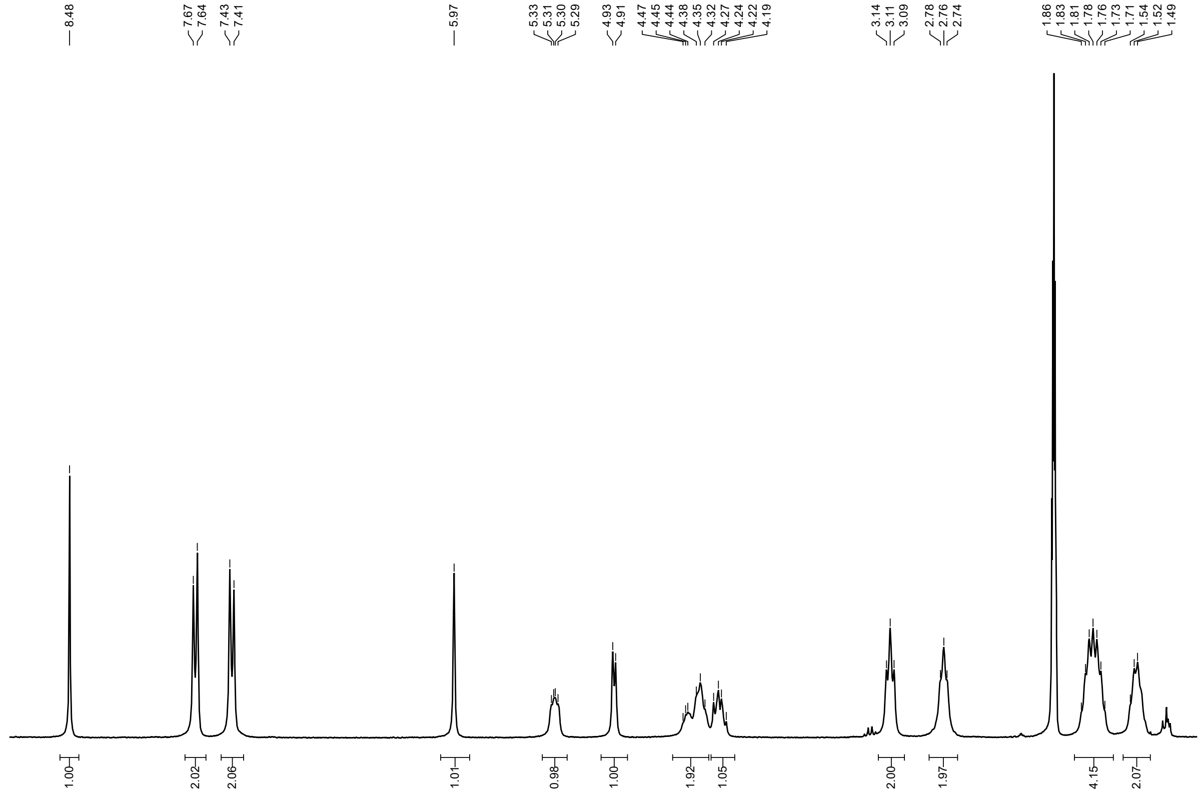

^{13}C NMR spectrum of compound **12** recorded in AcOH-d_4 at 300 K (75 MHz):

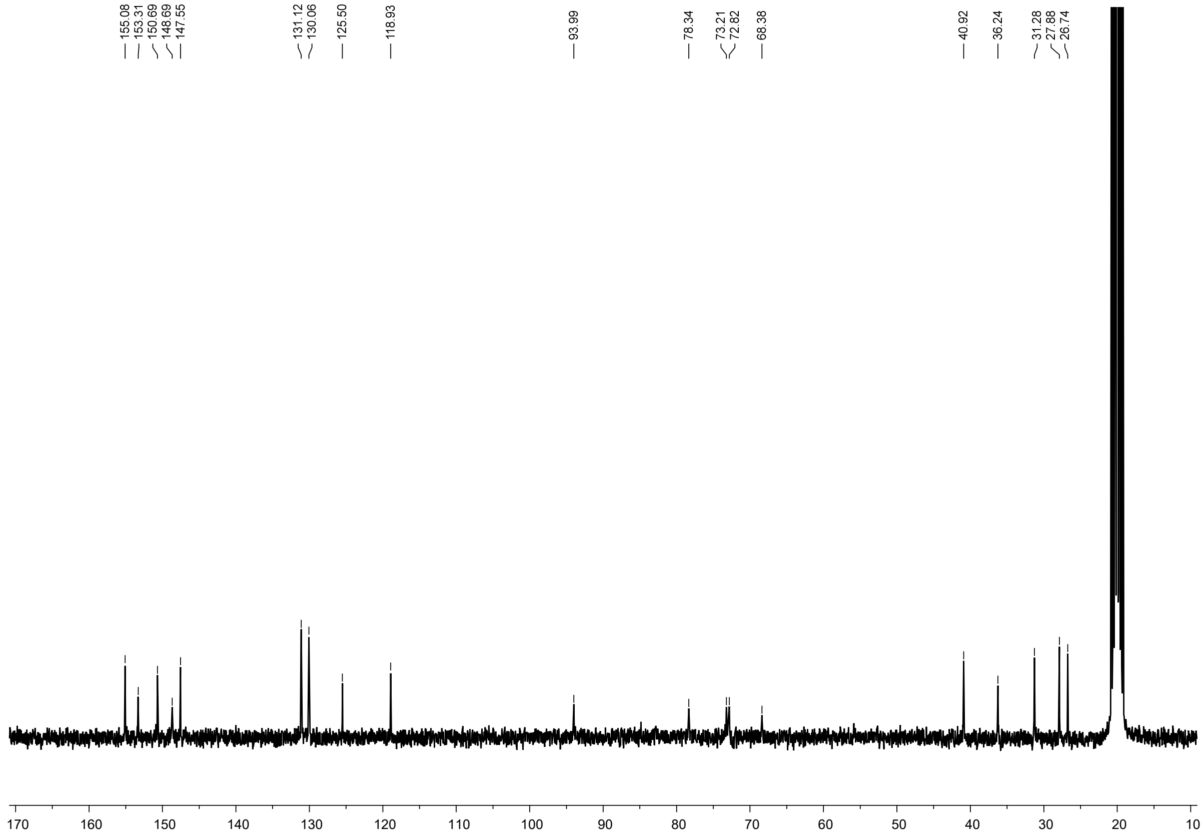

APCI-MS spectrum of compound **2**:

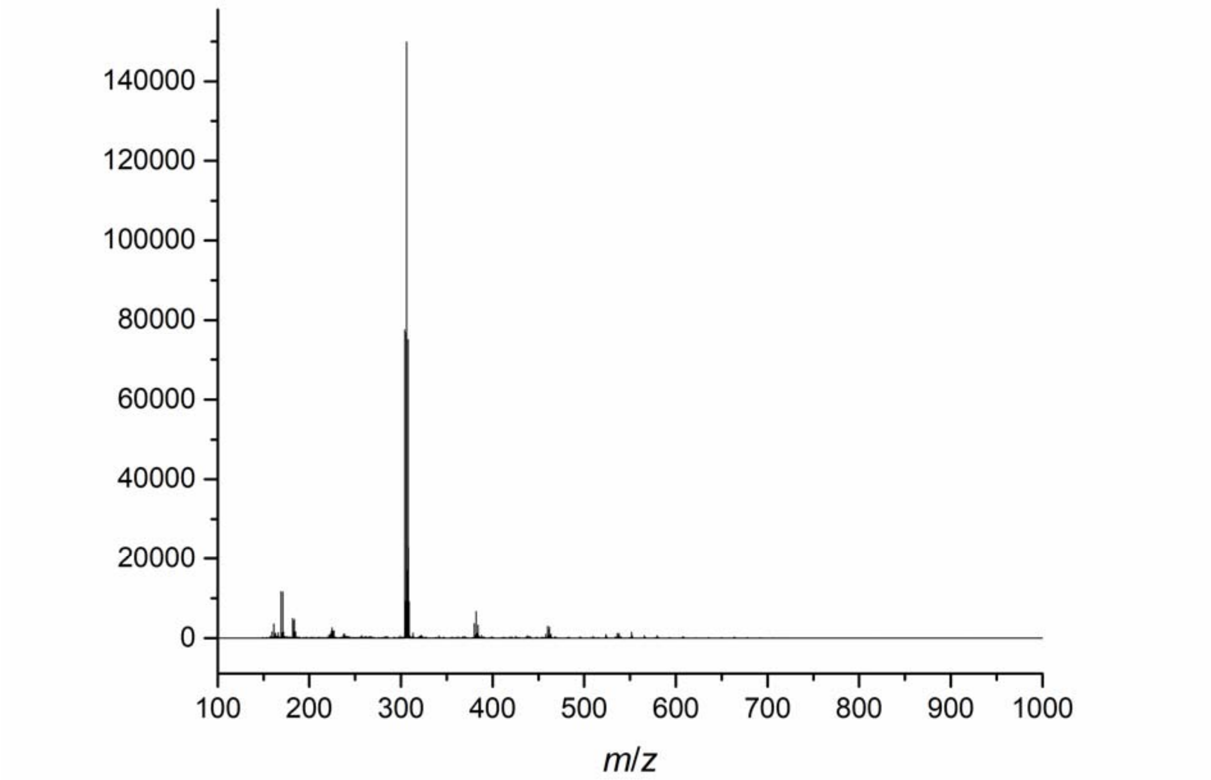

ESI-MS spectrum of compound **3**:

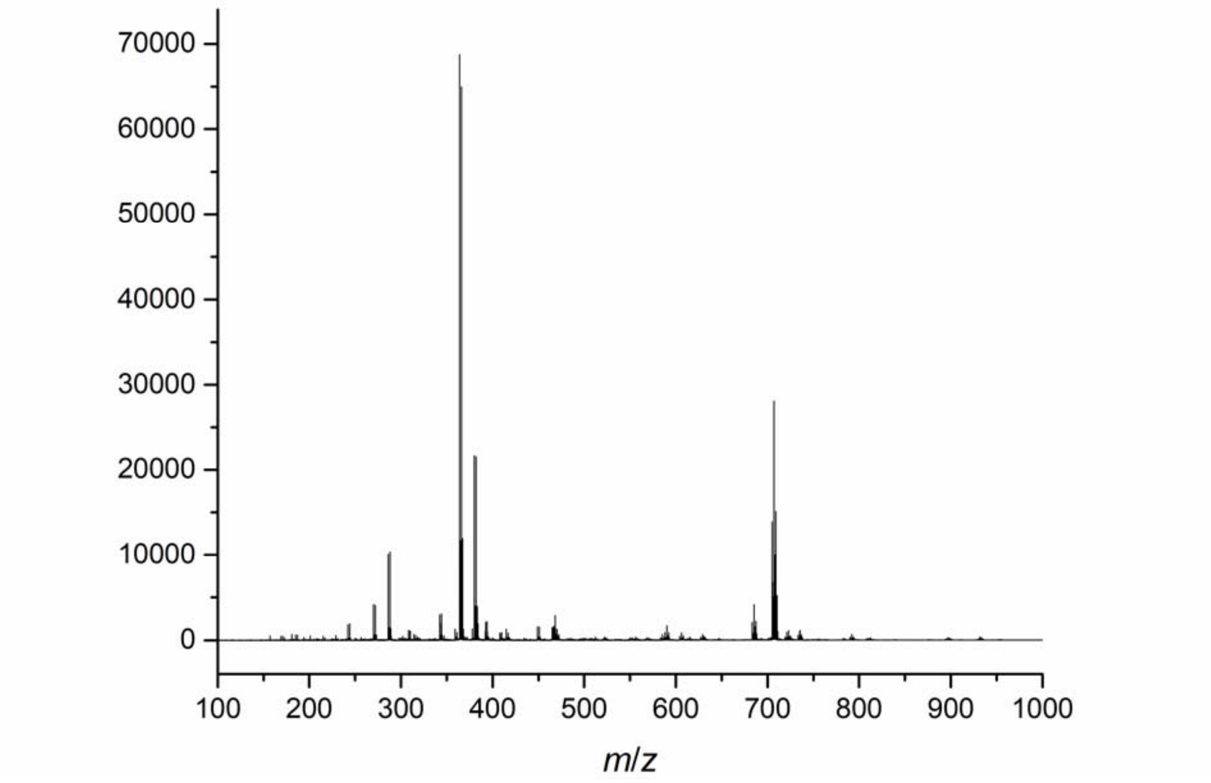

ESI-MS spectrum of compound **4**:

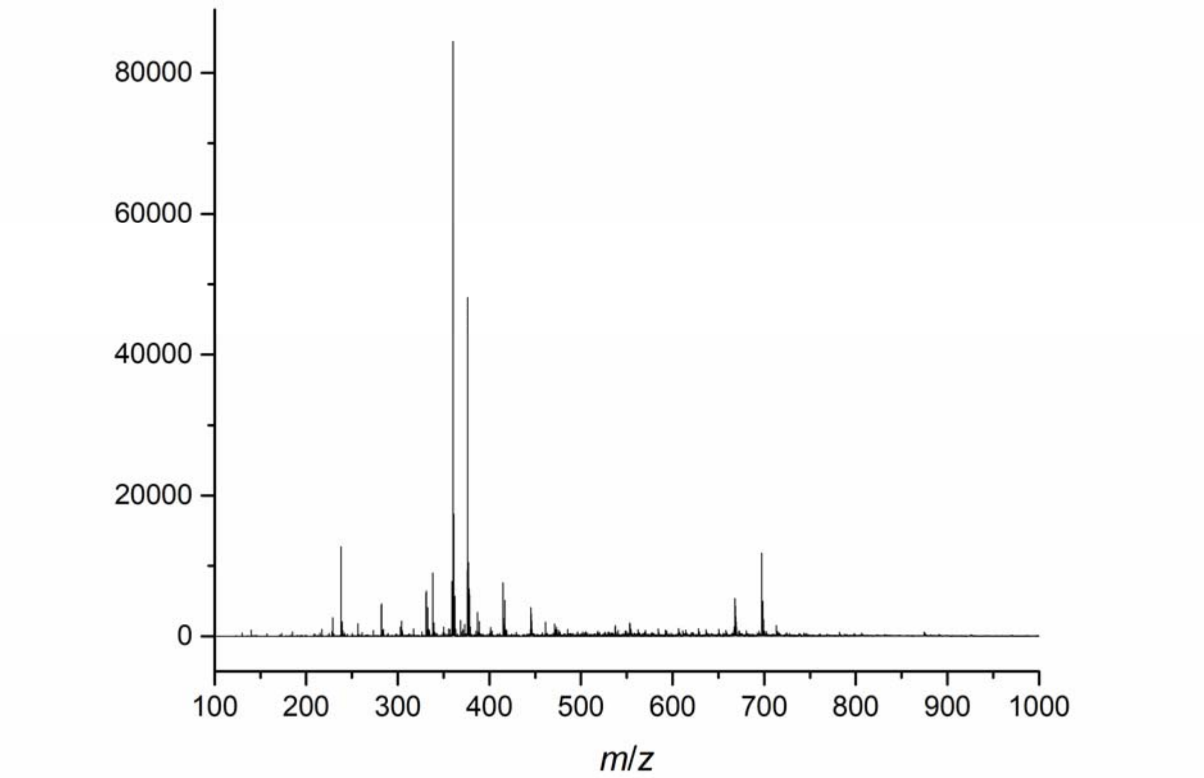

ESI-MS spectrum of compound **6**:

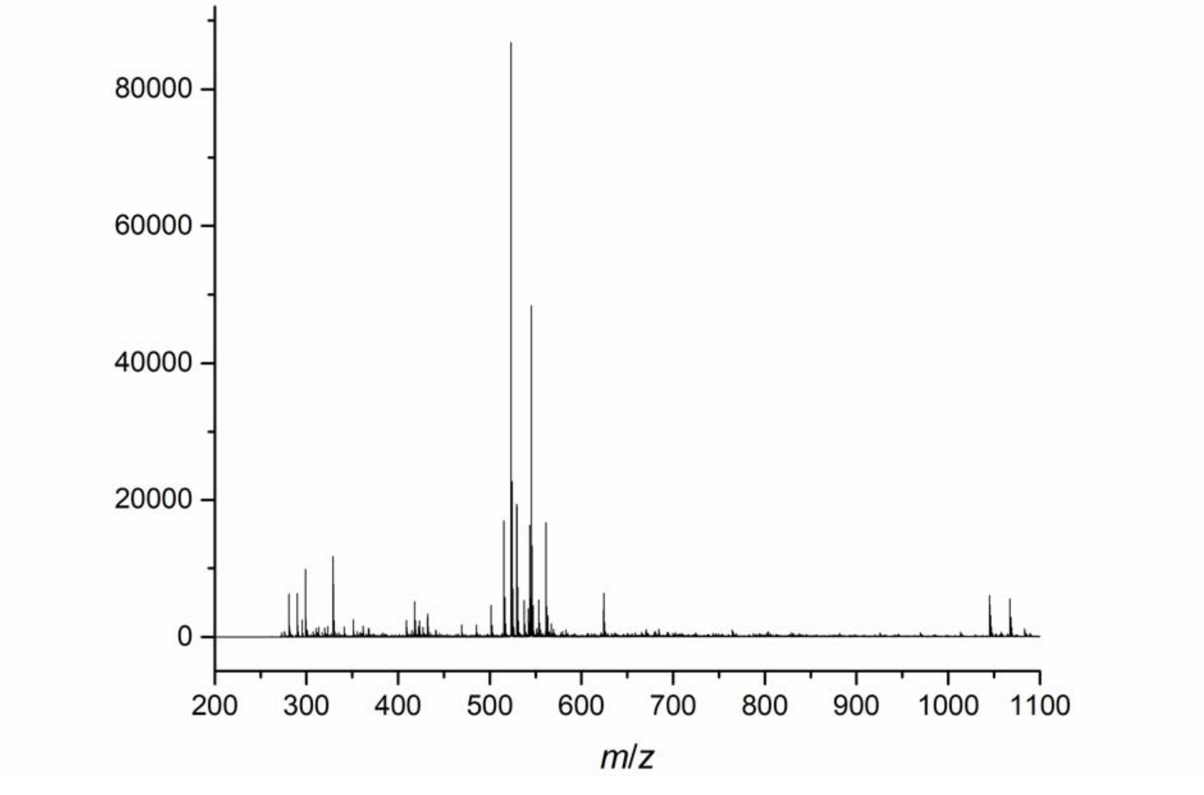

ESI-MS spectrum of compound **7**:

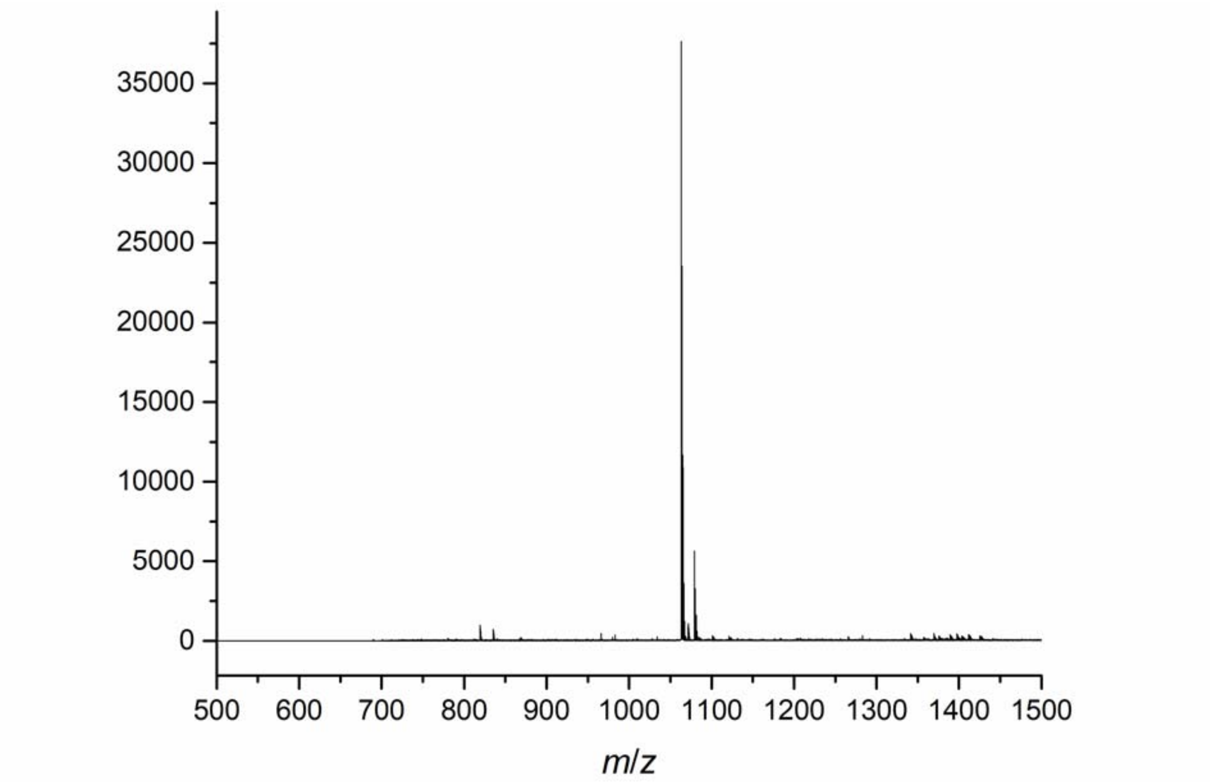

ESI-MS spectrum of compound **9**:

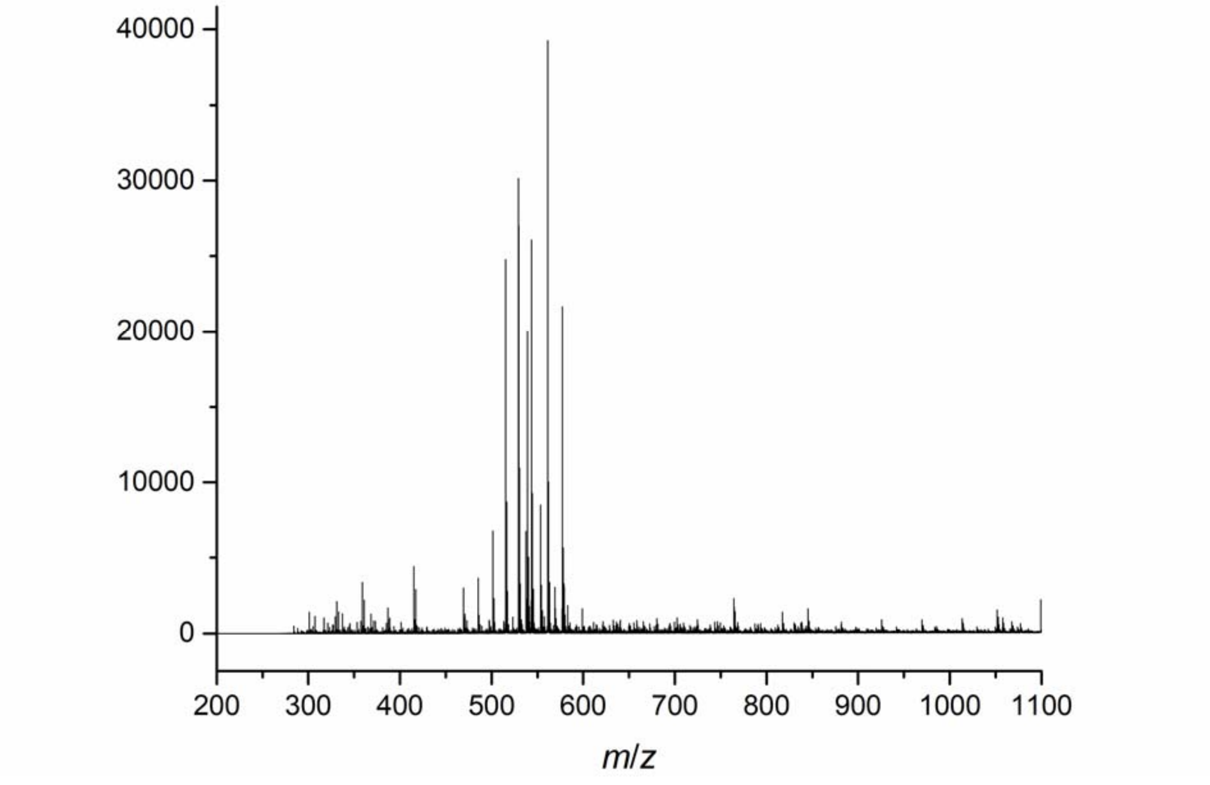

ESI-MS spectrum of compound **10**:

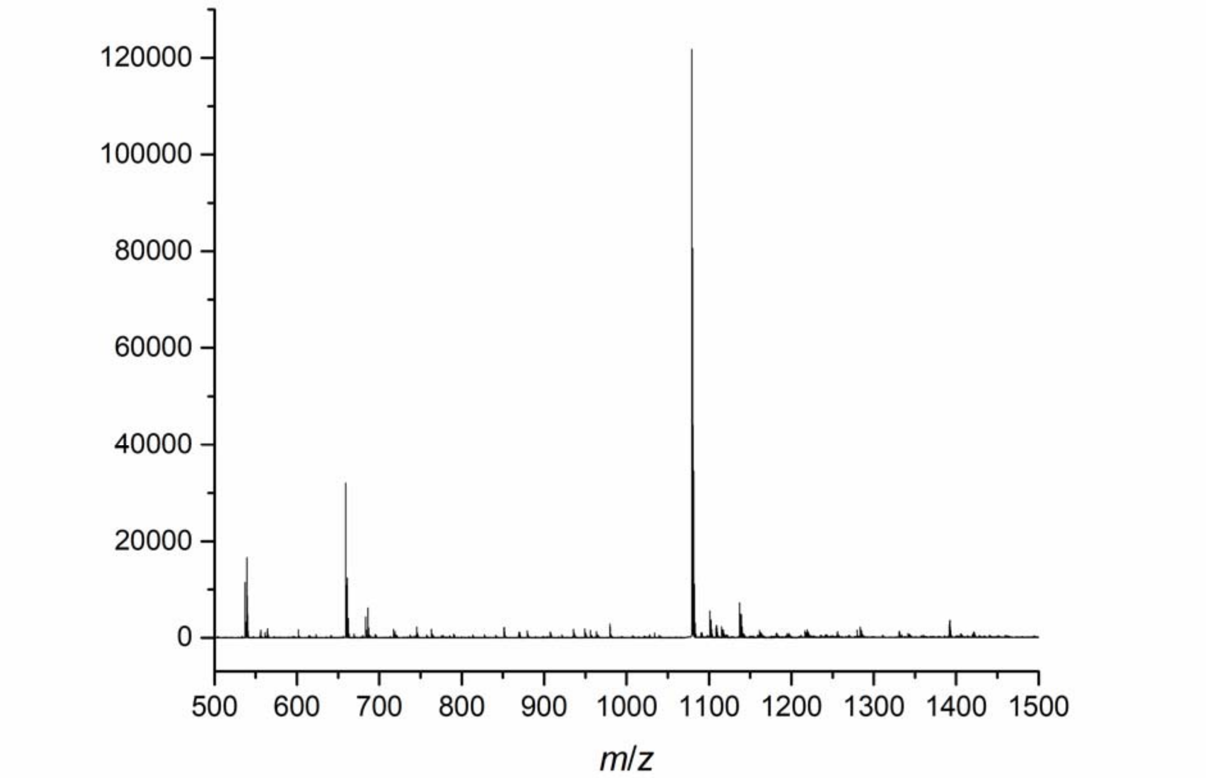

ESI-MS spectrum of compound **11**:

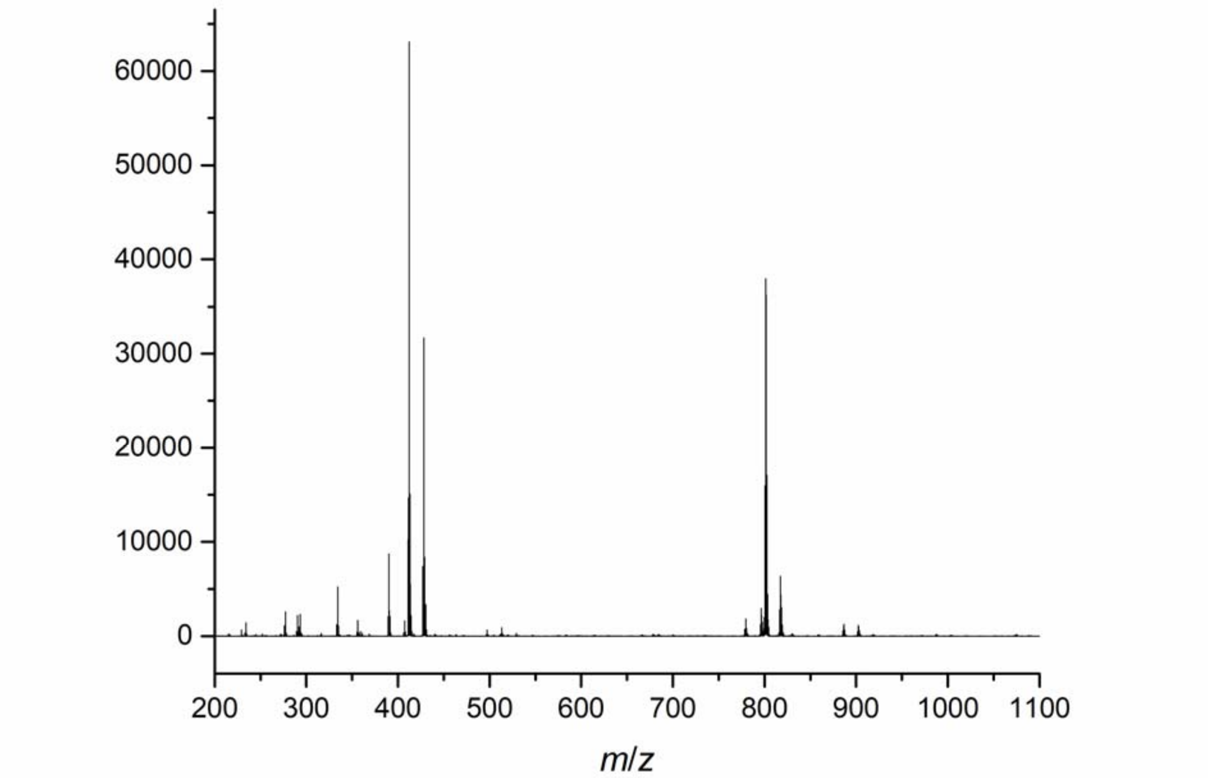

ESI-MS spectrum of compound **12**:

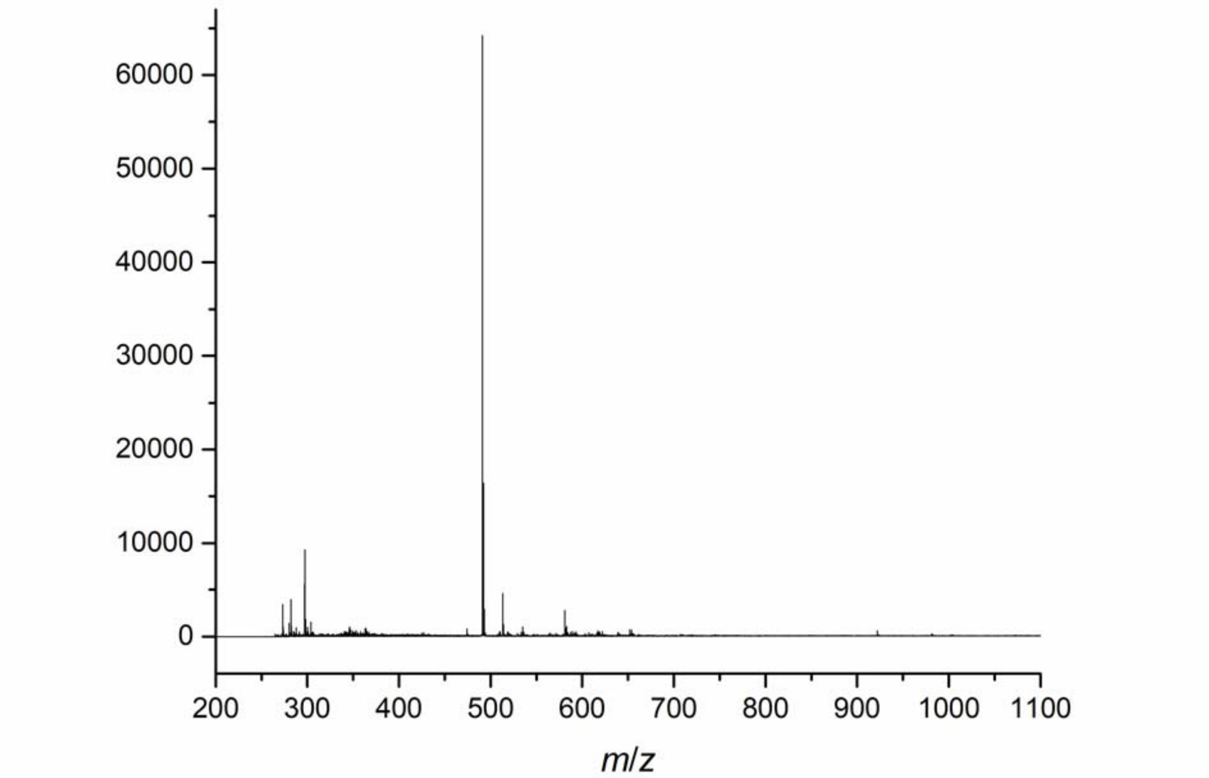

ESI-MS spectrum of compound **13**:

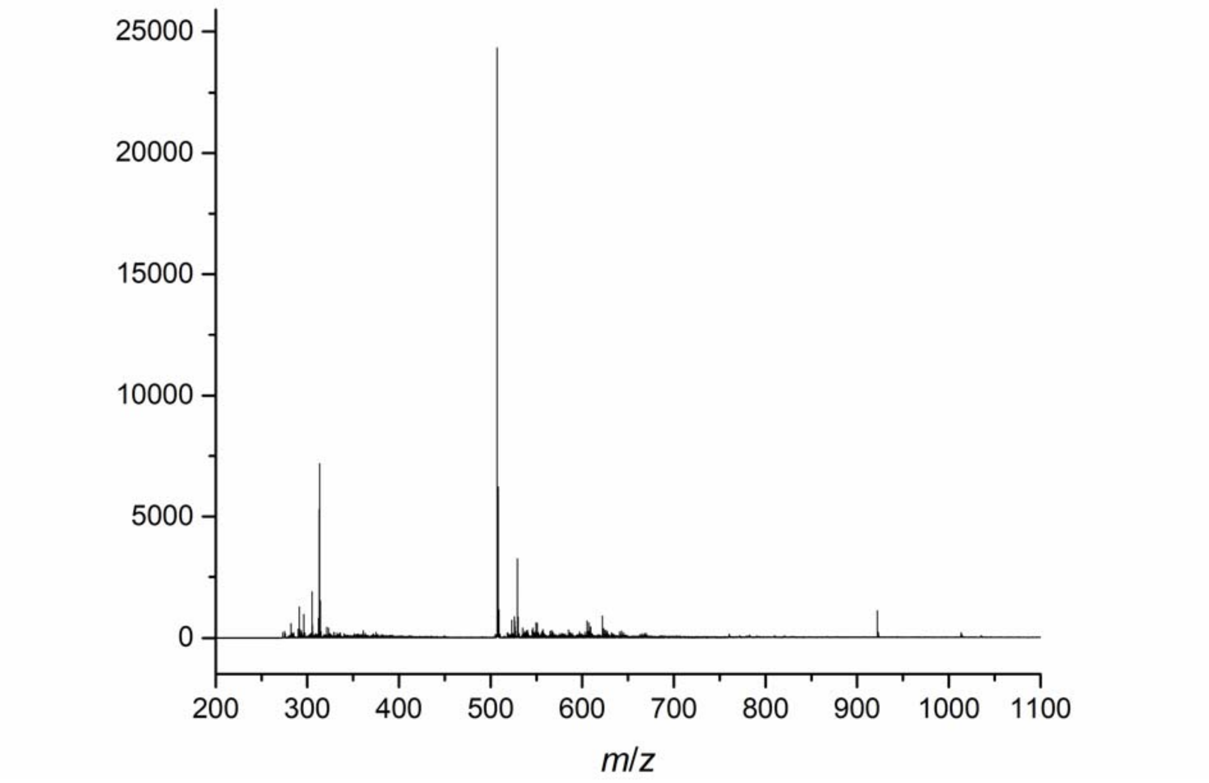

RP-HPLC chromatogram of compound **7** (t_R = 39.1 min):

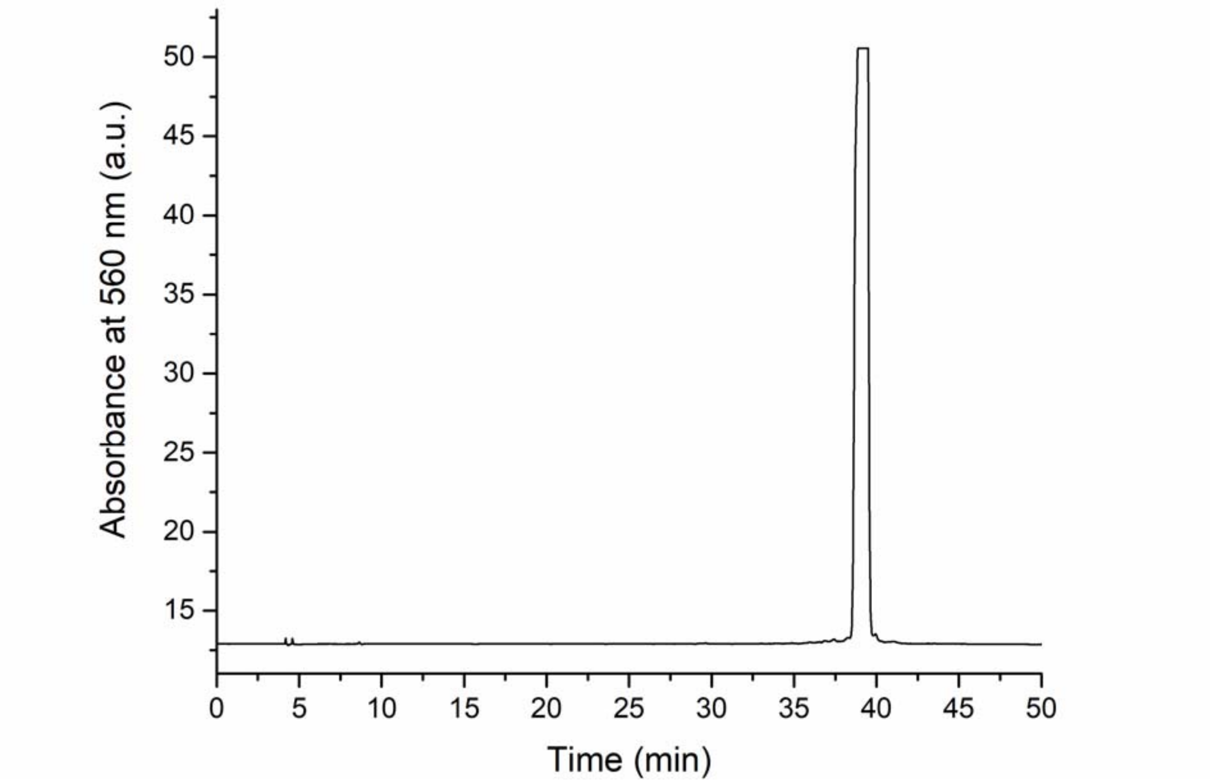

RP-HPLC chromatogram of compound **10** (t_R = 38.1 min):

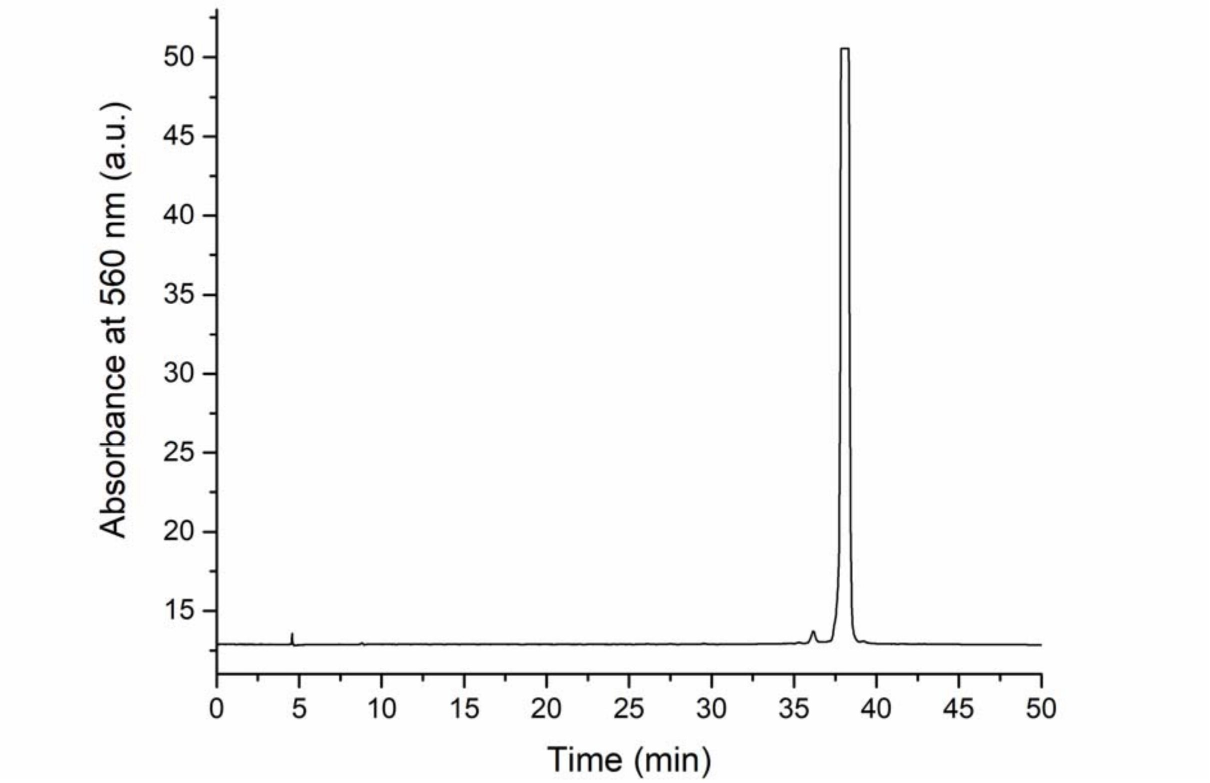

RP-HPLC chromatogram of compound **13** (t_R = 21.0 min):

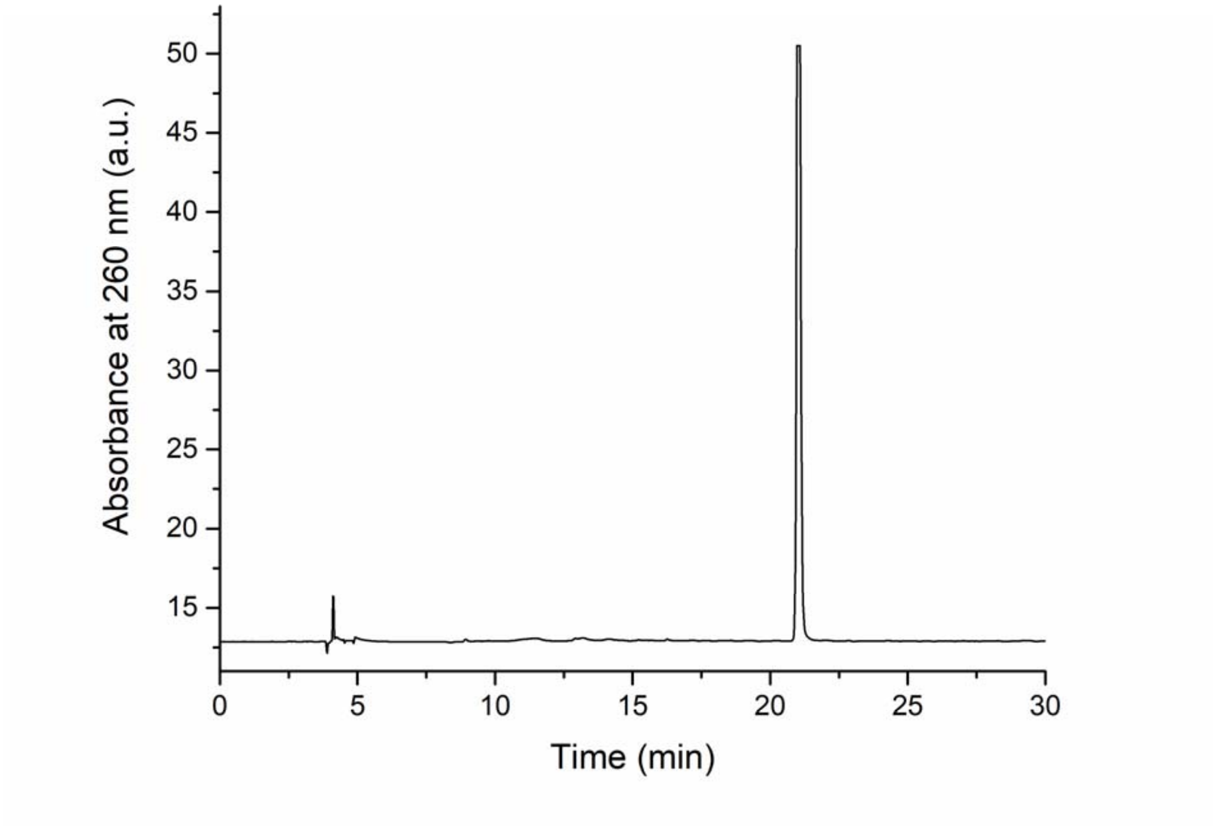

Details of the differences in the crystallographic binding mode of (A) cAMP and the predicted binding modes of cNMP derivatives (B) **13**, (C) **12**, (D) **6**, and (E) **9** in murine HCN2:

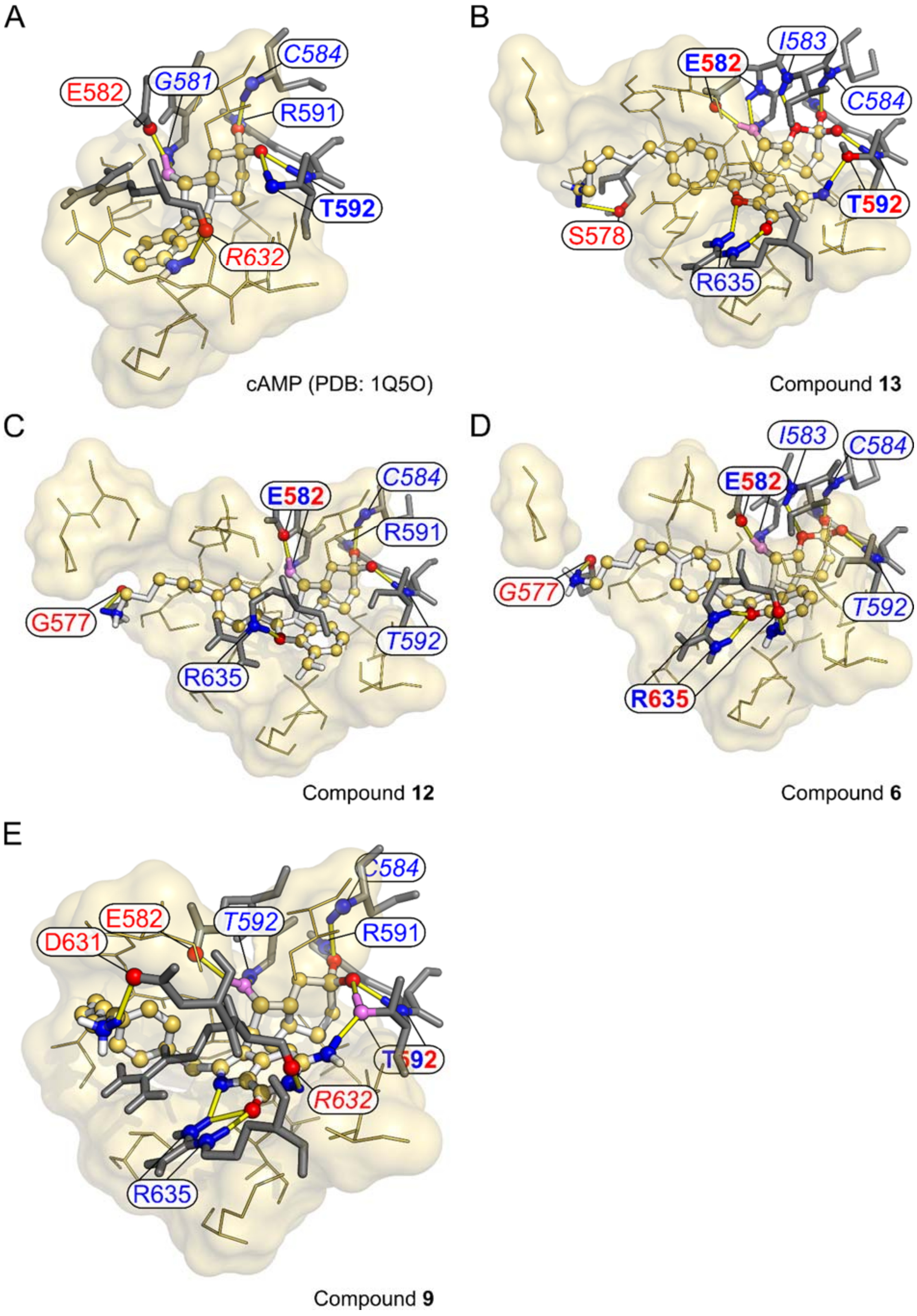

The ligand is depicted as white sticks. Ligand atoms interacting with the protein only through van der Waals contacts are depicted as gold spheres; the corresponding amino acids in the protein are depicted in gold line-and-surface representation. Amino acids that form hydrogen bonds are shown as grey sticks. The atoms involved in hydrogen bonds are colored according to their involvement as donor (blue), acceptor (red), or mixed donor-acceptor (violet) and are depicted as spheres. Amino acid labels are colored according to the involvement of the respective amino acid as donor (blue), acceptor (red), or mixed donor-acceptor (blue and red). Amino acids in which only the side chain is involved in the hydrogen bond are labeled in normal font style, those in which only the backbone is involved are labeled in italic font style, and those in which both side chain and backbone are involved are labeled in bold font.
